# The Association of Genetic Variants Within the Type XII Collagen and Tenascin C Genes with Knee Joint Laxity Measurements

**DOI:** 10.3390/genes16020164

**Published:** 2025-01-27

**Authors:** Samantha Beckley, Roopam Dey, Shaun Stinton, Willem van der Merwe, Thomas Branch, Alison V. September, Michael Posthumus, Malcolm Collins

**Affiliations:** 1 Health Through Physical Activity, Lifestyle and Sport Research Centre (HPALS), Division of Physiological Sciences, Department of Human Biology, University of Cape Town, Rondebosch, Cape Town 7700, South Africa; s.beckley@arthroresearch.com (S.B.); willem@ssoc.co.za (W.v.d.M.); alison.september@uct.ac.za (A.V.S.); michael.posthumus@uct.ac.za (M.P.); 2 Division of Biomedical Engineering and Division of Orthopaedic Surgery, Faculty of Health Sciences, University of Cape Town, Rondebosch, Cape Town 7700, South Africa; roopam.dey@uct.ac.za; 3 End Range of Motion Improvement, Atlanta, GA 30324, USA; s.stinton@ermi-motion.com (S.S.); doctorbranch@yahoo.com (T.B.); 4 Sports Science Orthopaedic Clinic, Sports Science Institute of South Africa, Newlands, Cape Town 7700, South Africa; 5 Sports Science Institute of South Africa, Newlands, Cape Town 7700, South Africa

**Keywords:** genu recurvatum, anterior–posterior tibial translation, external–internal tibial rotation, ligament, tendon, knee laxity

## Abstract

Background/Objectives: Types I, V, and XI collagen gene variants have been reported to associate with measurements of knee joint laxity and/or absolute knee ligament length changes. Type XII collagen and tenascin C are also ligament structural proteins whose expression is regulated by mechanical loading. This study investigated whether *COL12A1* and *TNC* variants are associated with knee laxity and/or ligament length changes. Methods: Genu recurvatum, anterior–posterior tibial translation, external–internal tibial rotation, and ligament length changes were measured in 128 healthy participants. They were genotyped for *COL12A1* (rs970547) and *TNC* (rs1061494, rs2104772, rs1138545). Results: Both the *COL12A1* AA and *TNC* rs1061494 TT genotypes were associated with decreased external (*p* = 0.007, *p* = 0.010) and internal (*p* = 0.025, *p* = 0.002) rotation, as well as slack (*p* = 0.033, *p* = 0.014), in the dominant leg. Both genotypes, together with sex, weight, and/or *COL1A1* genotypes, explained 26% and 32% of the variance in external and internal rotation, respectively. The *TNC* genotype, sex, and BMI explained 23% of the variance in slack. The *COL12A1* AA and the *TNC* rs1061494 TT genotypes were associated with smaller changes in the MCL (aMCL: *COL12A1 p* = 0.009, *TNC p* = 0.045; iMCL: *COL12A1 p* = 0.004, *TNC p* = 0.043; pMCL: *COL12A1 p* = 0.003, *TNC p* = 0.067; aDMCL: *COL12A1 p* = 0.007, *TNC p* = 0.020; pDMCL: *COL12A1 p* = 0.007, *TNC p* = 0.023) and/or LCL (*COL12A1 p* = 0.652, *TNC p* = 0.049) lengths within the dominant knee. The *TNC* rs1061494 CC genotype was associated with larger changes in the non-dominant anterior (*p* = 0.021) and posterior (*p* < 0.001) ACL bundle lengths. Conclusions: These findings suggest that *COL12A1* and *TNC* variants are associated with internal–external tibial rotation and knee ligament length changes in healthy individuals.

## 1. Introduction

The measured movement of a joint (laxity) is a multifactorial trait with a heritable component and is determined by the structure of its ligaments, bone shapes, and joint congruency [1,2]. Mutations within several collagen-encoding genes, such as *COL1A1* and *COL5A1*, cause connective tissue disorders with joint hypermobility presenting as one of the clinical symptoms [3]. More recently, common DNA sequence variants within the *COL1A1*, *COL5A1*, *COL11A1,* and *COL11A2* genes have been reported to associate with measurements of knee joint laxity [4,5,6].

The *COL1A1* rs1107946 GG genotype is associated with larger external and internal tibial rotation in the non-dominant limb, which is collectively resisted by the four major stabilising ligaments within the knee, namely the medial (MCL) and lateral (LCL) collateral ligaments, as well as the anterior (ACL) and posterior (PCL) cruciate ligaments [5]. The PCL and ACL also resist excessive posterior and anterior displacement (translation) of the tibia relative to the femur, respectively. The GG genotype at both the *COL1A1* rs1107946 and rs1800012 variants is associated with larger active and maximum tibial displacement, as well as larger changes in MCL and LCL length in the non-dominant limb [5]. Although Beckley and colleagues did not find an association [5], the *COL1A1* rs1800012 T allele has been reported to associate with increased knee hyperextension [4]. Allelic interactions between *COL5A1* rs12722, *COL11A1* rs3753841, *COL11A1* rs1676486, and *COL11A2* rs1799907 are associated with active and passive genu recurvatum of the non-dominat knee [6]. In line with this finding, the *COL5A1* rs12722 CC genotype was associated with decreased genu recurvatum within females [4]. These collagen genes encode specific α chains within types I, V, and/or XI collagen, which are structural components of the fibril within ligaments and other musculoskeletal connective tissues.

Type XII collagen, on the other hand, is located on the surface of the type I collagen fibril and is a homotrimer encoded by *COL12A1* [7,8]. Similarly, to the other collagen-encoding genes, *COL12A1* mutations have been implicated in inherited disorders with joint hypermobility as one of the symptoms [9,10]. Specifically, mutations within *COL12A1* cause myopathic Ehlers–Danlos syndrome (EDS) which presents with hypermobility of the distal joints [11]. The *COL12A1* rs970547 AA genotype has been associated with increased anterior displacement of the tibia relative to the femur in female participants [4]. Although not a member of the collagen family, tenascin C, which is encoded by *TNC*, plays a critical regulatory role in connective tissue biology [12]. Mutations in another member of the tenascin family, *TNXB*, encoding tenascin-X, cause classical-like EDS [13,14]. Since both *TNC* and *COL12A1* have been found to share a putative stretch-responsive enhancer region, their expression is upregulated by mechanical loading [15]. We therefore hypothesised that common *COL12A1* and *TNC* variants will also modulate measurements of knee joint laxity and/or computed knee ligament length changes using simulated knee modelling. The dominant leg undergoes more adaptation to repeated loading in comparison to the non-dominant leg [16]. Due to the presence of the stretch-responsive region within the *COL12A1* and *TNC* promoters, these associations were investigated in both the non-dominant and dominant legs.

Therefore, the aim of this study was to investigate whether *COL12A1* (rs970547, A/G) and *TNC* (rs1061494, T/C; rs1138545, C/T; rs2104772, T/A) variants are associated independently or via gene-gene interactions with genu recurvatum, anterior–posterior tibial displacement, and external–internal tibial rotation. An additional aim of the study was to determine whether these gene variants are significant contributors in multiple linear regression models of the knee joint laxity measurements, as well as any computed knee ligament length changes.

## 2. Materials and Methods

One hundred and twenty-eight moderately active, apparently healthy participants of self-reported European ancestry, with no history of spinal cord injury or any injury to at least one knee, were recruited between February 2016 and October 2018 from fitness centres within the greater Cape Town area in South Africa, as well as using social media and word of mouth as previously described to participate in a cross-sectional genetic association study [5,6]. Fourteen dominant (12 ACL, 1 MCL injury, and 1 other) and 8 non-dominant (5 ACL, 1 MCL, and 2 combined ACL and MCL injuries) knees with a history of injury were excluded from the analysis. Prior to testing, all participants were required to sign an informed consent form. Personal particulars (including leg dominance), sporting history, as well as previous medical and injury history questionnaires were completed by all participants.

A sit and reach test was used to assess the hip and lumbar range of motion, and the Beighton Score was used to assess generalised joint hypermobility (GJH) as previously described [2,17].

Passive and active genu recurvatum were measured in degrees as previously described, with lower degree values indicating increased hyperextension. [18]. Anterior and posterior translation (displacement) of the tibia relative to the femur, as well as maximum and active displacement, were measured as previously described [5,6]. External–internal rotation of the tibia, as well as slack, were measured using the robotic knee testing (RKT) device in a subset of the participants (*n* = 78) as previously described [19]. Briefly, the participants’ knees were clamped at 30° of flexion to the RKT device while they lay supine. The participants’ feet were attached firmly to servo motors that turned their tibia, hence the knee joint, externally until maximum external rotation at a toque of −5 Nm and then turned the joint internally until maximum internal rotation at a 5 Nm torque. Slack is defined as the amount of tibial rotation that occurred between the two turning points of the load deformation curve in the area of play between flanking external and internal rotation regions [19]. The reliability of these measurements has been previously published [5,19].

Each participant’s knee rotational angles, sampled for every 0.02 Nm increment in applied torque, were used to measure the length of ten knee ligament bundles during external to internal rotation using a previously validated Opensim (Stanford University, Stanford, CA, USA) lower limb musculoskeletal model consisting of 12 rigid bone segments, 23 degrees of freedom, 92 muscles, and 10 knee ligaments to measure [20]. The ligaments included two anterior cruciate ligament bundles (aACL and pACL), two posterior cruciate ligament bundles (aPCL and pPCL), three medial collateral ligament bundles (aMCL, iMCL, and pMCL), deep layer bundles of MCL (aDMCL and pDMCL), and a lateral collateral ligament bundle (LCL). Ligament lengths were defined as the distance between each ligament’s femoral and tibial attachment sites [20]. Absolute knee ligament length changes were calculated from maximum external and internal rotation [5,6,20].

A 5 mL venous blood sample was collected from the forearm of participants and stored at −20 °C until DNA extraction. DNA was extracted as previously described, and the DNA samples were stored at −20 °C [21]. DNA samples were genotyped for *COL12A1* rs970547 (A/G), *TNC* rs1061494 (T/C), *TNC* rs1138545 (C/T), and *TNC* rs2104772 (T/A) using pre-designed TaqMan™ Genotyping Assays (Applied Biosystems, Waltham, MA, USA). The polymerase chain reactions (PCR) were performed using the QuantStudio^®^ 3 Real-Time PCR System (Applied Biosystems), and the genotype calling, which was verified independently, was completed using the Genotyping Application on the Thermo Fisher Cloud (https://apps.thermofisher.com/apps/spa/, accessed on 1 April 2020). All plates included negative controls containing no DNA as well as additional positive controls with known genotypes.

The distribution of continuous data was analysed using Shapiro-Wilk normality tests. Normally distributed data were presented as mean and standard deviation and as median and interquartile ranges (IQR) if not normally distributed. Categorical data were presented as frequencies. Independent samples *t*-tests and Mann–Whitney-U tests were used to analyse parametric and non-parametric data, respectively, while Pearson Chi-Squared or Fisher’s Exact tests were used to analyse categorical data. Spearman’s rank-order correlations were used to assess any relationships between continuous physical characteristics and knee joint laxity measurements. Multiple linear regression model analyses using backward elimination were performed to test whether any of the physiological characteristics (sex, age, height, weight, and/or BMI) and genotypes were associated with knee joint laxity measurements. The IBM SPSS Statistics software (version 25) and/or Prism (version 10.0.0) was used for all remaining statistical analyses. Significance was set at *p* < 0.05. The rare *COL12A1* rs970547 GG genotype and the GA genotype were combined and compared to the AA genotype group during the analysis. Similarly, the rare *TNC* rs1138545 TT genotype and the CT genotype were combined and compared to the CC genotype group.

## 3. Results

### 3.1. General Characteristics

The median and interquartile ranges (Q1; Q3) of the 128 participants age, height, weight, and BMI were 26.0 (24.0; 32.0) years, 174.8 (167.0; 182.0) cm, 73.7 (61.1; 81.5) kg, and 23.7 (21.6; 25.7) kg/m^2^, respectively. Seventy-four (57.8%) were male, while 102 (79.7%) reported being right leg dominant, 5 (3.9%) reported being ambidextrous, and 71 (55.5%) reported taking part in flexibility training. All genetic variants were in Hardy-Weinberg Equilibrium (*COL12A1* rs970547 *p* = 0.928, *TNC* rs1061494 *p* = 0.960, *TNC* rs1138545 *p* = 0.700, *TNC* rs2104772 *p* = 0.816).

There were no significant differences in frequencies, medians, or means of the participants sex, age, height, weight, BMI, flexibility training, Beighton scores, or sit and reach measurements between the *COL12A1* rs970547 (Table S1), *TNC* rs1061494 (Table S2), *TNC* rs1138545 (Table S3), and *TNC* rs2104772 (Table S4) genotype groups of all participants or, with the exception of the *COL12A1* genotype groups, the subset of participants whose external and internal tibial rotation were measured. The subset of participants with a *COL12A1* rs970547 AA genotype had a statistically significant (*p* = 0.034) higher sit and reach measurement (46.1 ± 9.6 cm) compared to those with either an AG or GG genotype (G-allele carrier) (41.1 ± 10.5 cm). There were no other *COL12A1* genotype effects on the general characteristics of this subset of participants, their flexibility training, or Beighton scores (Table S1).

### 3.2. Association of COL12A1 and TNC Genotypes with Knee Laxity Measurements

**Figure 1:**
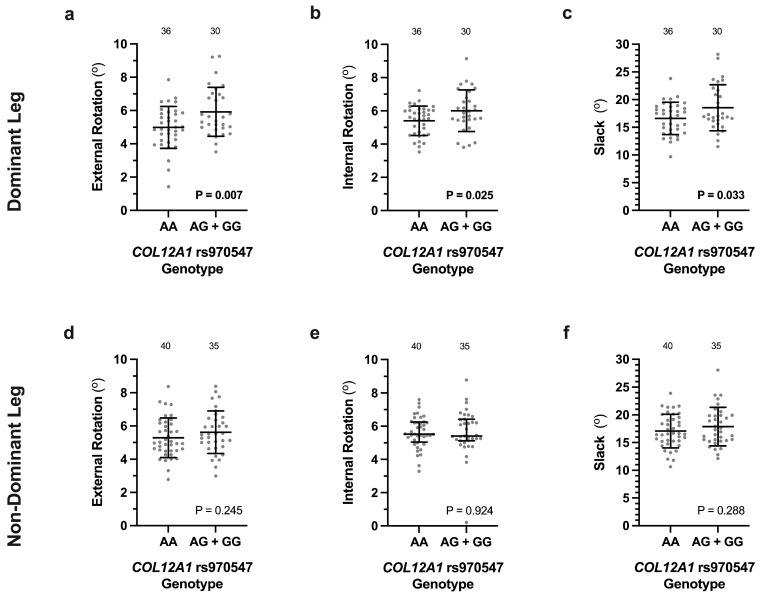
The *COL12A1* rs970547 (A/G) genotype effects on the external and internal tibial rotation, as well as slack, measured using the robotic knee testing (RKT) device of participants uninjured dominant (**a**–**c**) and non-dominant (**d**–**f**) legs. Expcet for internal rotation of the non-dominant knee (**e**), which is expressed as median (IQR), all other measurements are expressed as average ± standard deviation. The rare GG (dominant leg *n* = 4, non-dominant leg *n* = 5) genotype and the GA genotype were combined for the analysis. The individual values are shown as light grey circles, and the number of participants in each group is indicated. Unable to genotype 1 participant for rs970547. Significantly different *p* values are indicated in bold.

The average external (5.0 ± 1.3°, *p* = 0.007) and internal (5.4 ± 0.9°, *p* = 0.025) tibial rotation, as well as slack (16.6 ± 2.9°, *p* = 0.033), of the dominant leg of participants with a *COL12A1* rs970547 AA genotype (*n* = 36) were significantly lower than those with either an AG or GG genotype (G-allele carrier) (5.9 ± 1.5° external rotation; 6.0 ± 1.3° internal rotation; 18.5 ± 4.2° slack; *n* = 30) (Figure 1a–c). There were, however, no significant differences in external rotation (*p* = 0.254), internal rotation (*p* = 0.924), or slack (*p* = 0.288) between the *COL12A1* rs970547 genotype groups of the non-dominant leg (Figure 1d–f).

**Figure 2:**
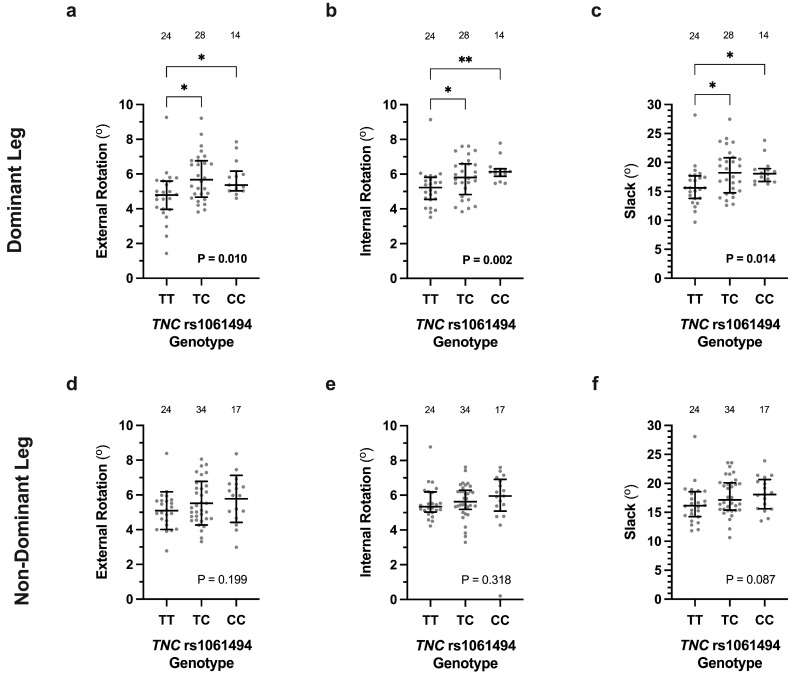
The *TNC* rs1061494 (T/C) genotype effects on the external and internal tibial rotation, as well as slack, measured using the robotic knee testing (RKT) device of participants uninjured dominant (**a**–**c**) and non-dominant (**d**–**f**) legs. Expcet for external rotation of the non-dominant knee (**d**), which is expressed as average ± standard deviation, all other measurements are expressed as median (IQR). The individual values are shown as light grey circles, and the number of participants in each group is indicated. Significantly different *p* values are indicated in bold with post-hoc analysis differences indicated with asterisks. * *p* < 0.05 and ** *p* < 0.001.

The median external [4.8 (4.0; 5.6)°, *p* = 0.010] and internal [5.2 (4.5; 5.8)°, *p* = 0.002] tibial rotation, as well as slack [15.6 (13.8; 17.7)°, *p* = 0.014], of the dominant leg of the participants with a *TNC* rs1061494 TT genotype (*n* = 24) were also significantly lower than those with either a TC [External Rotation 5.7 (4.7; 6.8)°; Internal Rotation 5.8 (4.8; 6.6)°; Slack 18.2 (14.8; 20.8)°; *n* = 28] or CC genotype [External Rotation 5.4 (5.0; 6.2)°; Internal Rotation 6.1 (5.9; 6.3)°; Slack 18.1 (16.7; 18.9)°; *n* = 14] (Figure 2a–c). There were, however, no significant differences in external rotation (*p* = 0.119), internal rotation (*p* = 0.318), or slack (*p* = 0.087) between the *TNC* rs1061494 genotype groups of the non-dominant leg (Figure 2d–f).

There were no *COL12A1* rs970547 and *TNC* rs1061494 genotype effects on any of the genu recurvatum or anterior–posterior translation measurements of either the dominant or non-dominant legs (Tables S5 and S6). Additionally, there were no *TNC* rs1138545 or rs2104772 genotype effects on any of the knee laxity measurements of the dominant and non-dominant legs (Tables S7 and S8).

### 3.3. Contribution of COL12A1, TNC and COL1A1 Genotypes to Multiple Linear Regression Models

Since *COL1A1* variants have previously been reported to associate with the non-dominant knee rotational laxity measurements [5], the association of *COL1A1* rs1107946 (G/T) and rs1800012 (G/T) with these measurements in the dominant knee was analysed. Neither of the *COL1A1* variants were independently associated with external (rs1107946 *p* = 0.842; rs1800012 *p* = 0.576) and internal (rs1107946 *p* = 0.189, rs1800012 *p* = 0.105) tibial rotation laxity, nor slack (rs1107946 *p* = 0.497; rs1800012 *p* = 0.199), of the dominant knee (Table S9). However, the average internal tibial rotation of the dominant knee was significantly higher (*p* = 0.025) when participants with a GG genotype at both *COL1A1* variants (6.0 ± 1.1°) were compared to those with the other remaining genotype combinations (5.4 ± 1.0°) (Table S10). In addition, females had on average significantly higher external tibial rotation (females: 5.9 ± 1.6° vs. males: 5.1 ± 1.2°, *p* = 0.022) and slack (females: 18.6 ± 4.1° vs. males: 16.8 ± 3.1°, *p* = 0.045) of the dominant leg. Both weight and BMI were negatively correlated with internal tibial rotation (weight: r = −0.271, *p* = 0.028; BMI: r = −0.389, *p* = 0.001) and slack (weight: r = −0.333, *p* = 0.006; BMI: r = −0.338, *p* = 0.006) of the dominant leg (Table S11).

The *COL12A1* rs970547 and *TNC* rs1061494 genotypes, as well as sex, were therefore significant predictors for the external tibial rotation, whereby the model explained 26% of the variance (Table 1). Specifically, the analysis showed that being a male, as well as having *COL12A1* AA and *TNC* TT genotypes, was predicted to decrease external rotational laxity by 0.74°, 0.80°, and 1.04°, respectively, when all of the other variables were held constant. Significant predictors of internal tibial rotation, which explained 32% of the variance, included *COL12A1*, *TNC,* and *COL1A1* genotypes, together with BMI (Table 1). A decrease in BMI by one unit, as well as the *COL12A1* AA and *TNC* TT genotypes, predicted a decrease in internal rotational laxity by 0.12°, 0.47°, and 0.69°, respectively, while a GG genotype at both *COL1A1* variants predicted an increase in internal tibial rotation by 0.63°. Finally, sex, BMI, and *TNC* genotype were significant predictors for the slack of the dominant knee, whereby the model explained 23% of the variance (Table 1). For males, a decrease in BMI by one unit and the *TNC* TT genotype predicted a decrease in slack by 1.71°, 0.38°, and 2.39°, respectively.

### 3.4. Ligament Length Changes

**Figure 3:**
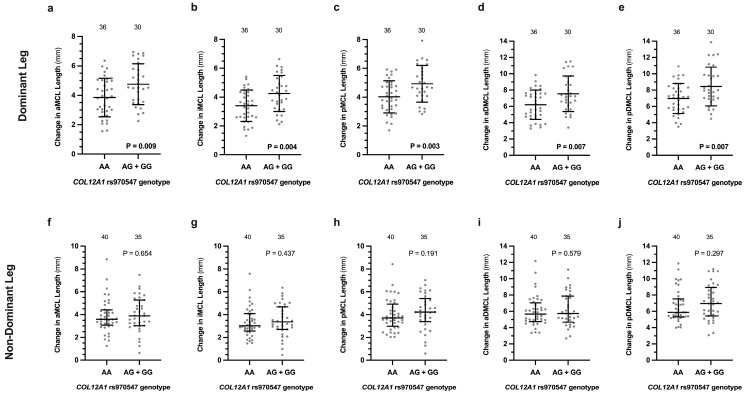
The *COL12A1* rs970547 (A/G) genotype effects on the absolute change in the calculated length of the (**a**,**f**) anterior bundle of the superficial layer of the MCL (aMCL), (**b**,**g**) inferior bundle of the superficial layer of the MCL (iMCL), (**c**,**h**) posterior bundle of the superficial layer of the MCL (pMCL), (**d**,**i**) anterior bundle of the deep layer of the MCL (aDMCL), and (**e**,**j**) posterior bundle of the deep layer of the MCL (pDMCL) during internal to external tibial rotation of the uninjured dominant (**a**–**e**) and non-dominant (**f**–**j**) legs. The dominant and non-dominant leg ligament length changes are expressed as average ± standard deviation and median (IQR) respectively. The rare GG (dominant leg *n* = 4, non-dominant leg *n* = 5) genotype and the GA genotype were combined for the analysis. The individual values are shown as light grey circles, and the number of participants in each group is indicated. Significantly different *p* values are indicated in bold.

When compared to the combined AG and GG genotypes (G-allele carriers), the *COL12A1* rs970547 AA genotype was associated with smaller absolute changes in ligament length during internal to external tibial rotation of all five MCL bundle namely the anterior superficial layer (AA: 3.9 ± 1.3 mm vs. G: 4.8 ± 1.4 mm, *p* = 0.009), inferior superficial layer (AA: 3.4 ± 1.1 mm vs. G: 4.3 ± 1.3 mm, *p* = 0.004), posterior superficial layer (AA: 4.0 ± 1.1 mm vs. G: 4.9 ± 1.3 mm, *p* = 0.003), anterior deep layer (AA 6.2 ± 1.8 mm vs. G 7.6 ± 2.2 mm, *p* = 0.007), and posterior deep layer (7.0 ± 1.8 mm vs. G: 8.4 ± 2.4 mm, *p* = 0.007), in the dominant leg (Figure 3a–e). There were, however, no significant differences in the absolute changes in length during external to internal tibial rotation of the non-dominant leg of the anterior superficial (*p* = 0.659), inferior superficial (*p* = 0.437), posterior superficial (*p* = 0.191), anterior deep (*p* = 0.579), and posterior deep (*p* = 0.297) MCL bundle layers (Figure 3f–j). There were also no significant differences in any of the lengths of the MCL bundles of the dominant and non-dominant legs during each period of external to internal rotation between the *COL12A1* genotype groups (Figures S1 and S2).

There were no significant differences in the changes of the anterior ACL (Dominant *p* = 0.068, Non-Dominant *p* = 0.837), posterior ACL (Dominant *p* = 0.352, Non-Dominant *p* = 0.673), anterior PCL (Dominant *p* = 0.954, Non-Dominant *p* = 0.202), posterior ACL (Dominant *p* = 0.431, Non-Dominant *p* = 0.747) and LCL (Dominant *p* = 0.652; Non-Dominant *p* = 0.829) lengths of either knee between the *COL12A1* genotype groups (Table S12).

Except for the posterior bundle of the superficial layer of the MCL (TT: 4.1 ± 1.1 mm vs. C: 4.7 ± 1.3 mm, *p* = 0.067), the *TNC* rs1061494 TT genotype groups had significantly smaller absolute changes in length of the anterior superficial (TT: 3.8 ± 1.3 mm vs. C: 4.5 ± 1.4 mm, *p* = 0.045), inferior superficial (TT: 3.4 ± 1.1 mm vs. C: 4.0 ± 1.3 mm, *p* = 0.043), anterior deep (TT: 6.0 ± 2.0 mm vs. C: 7.3 ± 2.0 mm, *p* = 0.020) and posterior deep (TT: 6.8 ± 2.0 vs. C: 9.1 ± 2.2 mm, *p* = 0.023) MCL bundle layers, as well as the LCL bundle [TT: 1.2 (0.9; 1.7) mm vs. C: 1.6 (1.0; 2.2) mm; *p* = 0.049], when compared to the combined TC and CC genotype groups in the dominant knee (Table S13). There were, however, no significant differences in the absolute changes in length during internal to external tibial rotation of the non-dominant leg of the anterior superficial (*p* = 0.142), inferior superficial (*p* = 0.214), posterior superficial (*p* = 0.284), anterior deep (*p* = 0.246), and posterior deep (*p* = 0.446) bundle layers of the MCL, as well as the LCL (*p* = 0.345) (Table S13). Except for the dominant anterior deep bundle of the MCL, which was significantly different between 10% and 30% of the motion (*p* = 0.021) (Figure S3), there were no other significant differences in the lengths of the MCL and LCL bundles of the dominant and non-dominant legs during each period of external to internal rotation between the *TNC* rs1061494 genotype groups (Figures S3 and S4).

There were no significant differences in the changes of the anterior ACL (Dominant *p* = 0.145, Non-Dominant *p* = 0.317), posterior ACL (Dominant *p* = 0.381, Non-Dominant *p* = 0.491), anterior PCL (Dominant *p* = 0.282, Non-Dominant *p* = 0.987) and posterior ACL (Dominant *p* = 0.224, Non-Dominant *p* = 0.195) lengths of either knee between the *TNC* TT genotype and C-allele carrier groups (Table S12).

**Figure 4:**
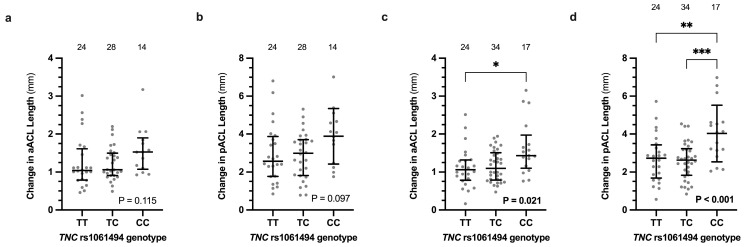
The *TNC* rs1061494 (T/C) genotype effects on the absolute change in the calculated length of the (**a**,**c**) anterior bundle of the ACL (aACL) and (**b**,**d**) posterior bundle of the ACL (pACL) during internal to external tibial rotation of the uninjured (**a**,**b**) dominant and (**c**,**d**) non-dominant legs. The aACL length changes are expressed as median (IQR), while the pACL length changes are expressed as average ± standard deviation. The individual values are shown as light grey circles, and the number of participants in each group is indicated. Significantly different *p* values are indicated in bold with post-hoc analysis differences indicated with asterisks. * *p* < 0.05, ** *p* < 0.001, and *** *p* < 0.0001.

**Figure 5:**
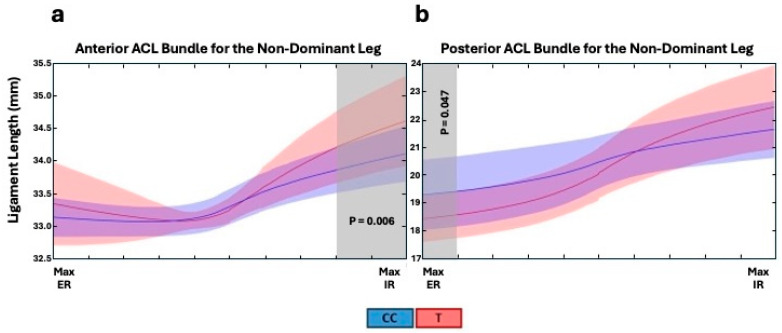
The *TNC* rs1061494 (T/C) genotype effects on the (**a**) anterior and (**b**) posterior ACL bundles lengths within the non-dominant leg from maximum (Max) external (ER) to maximum internal (IR) rotation. ACL bundle lengths were calculated at every 0.02 Nm increment in applied torque during each participant’s knee rotational angles from maximum external (−5 Nm torque) to maximum internal (5 Nm torque). The average (solid line) ligament length and standard deviations (shaded area) for the *TNC* rs1061494 CC genotype (CC) and combined TC and TT genotypes (T) are shown in blue and red, respectively, with areas of overlap represented in purple. The regions of significant differences in ligament bundle length are shaded.

There were, however, no significant changes in the length of the MCL bundles or LCL when all three genotypes were analysed (Table S14). Interestingly, the opposite *TNC* rs1061494 CC genotype was associated with larger changes in ligament length during internal to external tibial rotation of both ACL bundles (anterior ACL–TT: 1.1 [0.8; 1.3] mm; TC: 1.1 [0.8; 1.5] mm; CC: 1.4 [1.1; 2.0] mm; *p* = 0.021 and posterior ACL–TT: 2.7 ± 1.3 mm; TC: 2.6 ± 1.0 mm; CC: 4.0 ± 1.5 mm; *p* < 0.001) of only the non-dominant knee (Figure 4). Specifically, the *TNC* rs1061494 CC genotype was associated with increased anterior and posterior ACL bundle lengths at different periods of knee rotation of the non-dominant knee (Figure 5 and Figure S5). The significant difference was observed at maximum internal (80% to 100% of the motion; *p* = 0.006) and maximal external (0% to 9% of the motion; *p* = 0.047) rotation for the anterior ACL and posterior ACL bundles, respectively (Figure 5).

Finally, there were no significant differences in knee ligament length changes during internal to external tibial rotation of either the dominant or non-dominant legs between the *TNC* rs2104772 or rs1138545 (Tables S15 and S16), nor in the knee ligament length changes of the dominant knee between the combined *COL1A1* rs1107946 (G/T) and rs18000012 (G/T) (Table S17), or in the genotype groups.

## 4. Discussion

The main novel finding of this study was that *COL12A1* rs970547 AA and *TNC* rs1061494 TT genotypes were independently associated with decreased measurements of external and internal tibial rotation, as well as slack, in only the dominant leg. The GG genotype at both *COL1A1* rs1107946 and rs1800012 variants was independently associated with increased internal, but not external, tibial rotation of the dominant leg. Furthermore, all genotypes, together with BMI, were found to be significant predictors of the external tibial rotation measurement, while only the *COL12A1* and *TNC* genotypes, as well as sex, were significant predictors of the internal measurement in multiple linear regression models of the dominant leg. The *TNC* rs1061494 TT genotype, sex, and BMI were significant predictors of slack within the dominant knee. In agreement with these results, the *COL12A1* AA and *TNC* rs1061494 TT genotypes were associated with smaller absolute MCL and/or LCL length changes during internal to external rotation of only the dominant knee. Specifically, the *COL12A1* variant was associated with MCL length changes of all five bundles, while, expcet for the pMCL bundle, *TNC* rs1061494 was associated with MCL and LCL length changes in the remaining bundles. Only a significant difference between the 10% to 30% period of the aDMCLs motion was observed in the *TNC* rs1061494 genotype groups. In this study, the *TNC* rs1061494 CC genotype was associated with larger absolute length changes during external–internal tibial rotation of both ACL bundles in the non-dominant, but not the dominant, knee.

Although the *COL12A1* and *TNC* genotypes were not associated with measurements of external–internal tibial rotation in the non-dominant leg, we have previously reported that the *COL1A1* rs1107946 GG genotype had significantly larger tibial rotation measurements [5]. The GG genotype at both *COL1A1* rs1107946 and rs1800012 was also previously associated with larger absolute changes in MCL and LCL length in the non-dominant leg [5]. In this study, the combined *COL1A1* genotypes were, however, not associated with any absolute knee ligament length changes of the dominant limb. Although there are differences between the dominant and non-dominant legs, the current and previous results suggest that the absolute MCL and LCL length changes explain, at least in part, the association of the investigated *COL1A1*, *COL12A1,* and/or *TNC* variants with the external–internal tibial rotation measurements. Larger MCL length changes are more likely to occur in comparison to the ACL, which acts as a secondary restraint, during external rotation [22]. Contradictory findings of the LCL and other knee structures’ role in rotational stability have, however, been reported [23].

The collagen fibril, which consists predominantly of heterotrimeric type I collagen molecules and can also contain homotrimeric type I collagen molecules, is the basic structural component of ligaments and other non-cartilaginous musculoskeletal connective tissues. It has been proposed that the mechanical properties of these tissues are dependent of the relative proportion on the minor homotrimeric type I collagen molecules they contain. The *COL1A1* gene encodes for the α1(I) chains of both the hetero- and homotrimeric isoforms of type I collagen. The two investigated functional *COL1A1* variants are located within regulatory regions that have been hypothesised to modulate the amount of the homotrimer that is synthesised [24]. Transcriptional and post-transcriptional regulatory mechanisms are complex, and further research is therefore required to determine the functional role of these and other variants that can modulate the collagen fibril’s structure and the functional properties of ligaments. When all the published studies that included European populations were combined and analysed, the *COL1A1* rs1800012 TT genotype was previously associated with decreased risk of ACL ruptures [25]. Tibial rotation measurements and changes in knee ligament length do not mean that these variants cause these phenotypes. Any possible association between collagen gene variants, knee ligament length changes, tibial rotation measurements, and/or injury, such as ACL rupture, needs to be thoroughly investigated in the future. Specifically, future research should investigate whether the *COL1A1* variants modulate, at least in part, the MCL and LCL biomechanical properties, which result in larger length changes during rotational movements, resulting in greater strain on the ACL and therefore at increased risk of rupture.

The fibril-associated type XII collagen is functionally important in the development and growth of ligaments and tendons, as well as in tissue repair and regeneration [26,27]. Deficiency of this protein has been reported to impair tendon structure and function, as well as associate with increased risk of ACL rupture [26,28]. Different isoforms of type XII collagen are produced as a result of variants within the amino NC3 and carboxy NC1 domains with distinct temporal and tissue-specific expression patterns [27]. Several studies have reported that type XII collagen gene expression is upregulated by mechanical loading and that specific isoforms are predominately expressed in ligaments and tendons in response to mechanical loading [29,30]. Although the *COL12A1* rs970547 variant (A/G) is non-synonymous, resulting in the change of a serine to a glycine, the functional significance is currently unknown. It has, however, been proposed that the variant may alter the biochemical properties of the collagen fibril and, by implications, ligaments and other musculoskeletal connective tissues [31]. Previous bioinformatic analyses using the SIFT, PolyPhen-2, and FATHMM_ prediction tools described the Gly3058Ser substitution as moderately damaging [32]. Further research is therefore required to define the possible role of *COL12A1* rs970547 in connective tissue structural and biomechanical properties.

In adults, tenascin-C is primarily found in tissues that undergo high tensile loads after mechanical stress or injury [33,34]. Furthermore, it has also been hypothesised to play a role in determining the stiffness of tissues [35] as well as cell adhesion, migration, and proliferation [12]. Tenascin-C expression has specifically been noted to be increased in the synovial fluid of the knee after injury, surgery, or in diseased joints and is thought to be a marker of cartilage degradation [36]. The *TNC* rs1061494 (T/C) variant results in a non-synonymous change from glutamine to arginine within the first fibronectin type III repeat domain (FNIII) of the protein. Using bioinformatics tools, it has also been predicted that the CC genotype of this non-synonymous variant, which has also been associated with a 2.5-fold increased risk of chronic Achilles tendinopathy, may result in increased *TNC* expression [37]. Taken collectively, we hypothesise that the *TNC* rs1061494 variant may affect *TNC* expression in ligaments and tendons and thereby influence the mechanical properties of these structures and, in turn, with respect to the knee, affect the normal inter-individual variation in measurements of external–internal tibial rotation. Again, further research is required to test this hypothesis.

Since the association of the *COL12A1* and *TNC* variants with external–internal tibial rotation measurements and ligament length was observed in the dominant leg, it is tempting to hypothesise this may be as a result of an adaptation process caused by additional loading of the dominant leg, which may cause upregulation of expression of these genes, resulting in genotype-specific differences in the rotational measurements between the dominant and non-dominant legs. Supporting this hypothesis, Davis proposed in 1867 that biological soft tissues such as ligaments and tendons respond to increased loads by increasing the length and volume of such materials [38]. Moreover, previous studies have suggested that different adaptation occurs in the dominant leg versus the non-dominant leg, resulting in different changes in the musculoskeletal soft tissue in each of the legs [39,40].

A limitation of the study was that the rare homozygous genotypes had to be combined with the heterozygous genotypes during the analysis, specifically the GG and AG genotypes of *COL12A1* rs970547. It is possible that the reported association the *COL12A1* AA genotype and G allele with measurements of knee joint laxity and absolute knee ligament length changes is more complex. Future research should therefore investigate the possible independent association of the rare homozygous GG genotype. In addition, joint laxity is a multifactorial trait, and it is unlikely that only a few genes contribute to its genetic component. Therefore, future research should also investigate the possible contribution of variants within genes that encode structural components and regulators of biological processes within joint tissues. In addition to ligament structure, joint laxity is also determined by the structure of its bones and congruency of the joint. Future research should also consider the possible association of the investigated genetic variations on bone morphology and other anatomical features of the knee. A limitation of the OpenSim knee model was that subject-specific bone geometry or model scaling was not considered for recreating the musculoskeletal models for each participant. Sex is known to play a role in joint laxity and should be considered in future studies [41]. Specifically, females were not all tested at the same phase of the menstrual cycle [42].

## 5. Conclusions

In conclusion, the findings of this study demonstrated a potential role of the *TNC*, *COL12A1,* and *COL1A1* genes, specifically rs1061494, rs970547, rs1107946, and rs1800012, in modelled external–internal tibial rotation and the resulting knee ligament length changes in the dominant leg. The finding of this work may assist in determining the biological mechanisms and inter-individual variations of common musculoskeletal injuries, such as ACL ruptures and performance traits.

## Figures and Tables

**Table 1:** Summary of the multiple linear regression model for measurements of knee joint external and internal tibial rotation, as well as slack of the dominant leg.

		Estimate	Standard Error	95% CI	*p*-Value	
External Tibial Rotation (°)	Constant	6.66	0.30	6.07 to 7.26	<0.001	R^2^ = 0.29 Adjusted R^2^ = 0.26 *p* < 0.001
	Sex (Male)	−0.74	0.31	−1.36 to −0.12	0.021	
	*COL12A1* rs970547 (AA)	−0.80	0.31	−1.41 to −0.19	0.011	
	*TNC* rs1061494 (TT)	−1.04	0.32	−1.67 to −0.41	0.002	
Internal Tibial Rotation (°)	Constant	8.78	0.96	6.87 to 10.70	<0.001	R^2^ = 0.36 Adjusted R^2^ = 0.32 *p* < 0.001
	BMI (kg·m^−2^)	−0.12	0.04	−0.20 to −0.04	0.004	
	*COL12A1* rs970547 (AA)	−0.47	0.23	−0.94 to 0.00	0.049	
	*TNC* rs1061494 (TT)	−0.69	0.23	−1.16 to −0.22	0.005	
	*COL1A1* (GG and GG)	0.63	0.23	0.18 to 1.09	0.007	
Slack (°)	Constant	28.7	3.3	22.1 to 35.3	<0.001	R^2^ = 0.27 Adjusted R^2^ = 0.23 *p* < 0.001
	Sex (male)	−1.71	0.80	−3.31 to −0.10	0.037	
	BMI (kg·m^−2^)	−0.38	0.14	−0.65 to −0.11	0.006	
	*TNC* rs1061494 (TT)	−2.39	0.82	−4.02 to −0.75	0.005	

The physical characteristics, sex, weight and/or BMI, as well as *COL12A1* rs970547 (AA genotype and G allele), *TNC* rs1061494 (TT genotype and C allele), and/or the combined *COL1A1* rs1107946 and rs1800012 genotypes (GG + GG and rest of the genotype combinations) were considered for all models. Units or genotypes are shown in parentheses. Slack describes the amount of rotation that occurred between the two turning points of the curve in the area of play between flanking regions of external and internal rotation [19]. BMI, body mass index; CI, confidence interval; R^2^, coefficient of determination.

## Data Availability

The raw data supporting the conclusions of this article will be made available by the corresponding author on request.

## Supplementary Materials

The following supporting information can be downloaded at: https://www.mdpi.com/article/10.3390/genes16020164/s1, Table S1: The *COL12A1* rs970547 (A/G) genotype effects on all the participants general characteristics, Beighton score and sit and reach measurements, as well as the subset of participants that had their external and internal tibial rotation measured using the robotic knee testing (RKT) device; Table S2: The *TNC* rs1061494 (T/C) genotype effects on all the participants general characteristics, Beighton score and sit and reach measurements, as well as the subset of participants that had their external and internal tibial rotation measured using the robotic knee testing (RKT) device; Table S3: The *TNC* rs1138545 (C/T) genotype effects on all the participants general characteristics, Beighton score and sit and reach measurements, as well as the subset of participants that had their external and internal tibial rotation measured using the robotic knee testing (RKT) device; Table S4: The *TNC* rs2104772 (T/A) genotype effects on all the participants general characteristics, Beighton score and sit and reach measurements, as well as the subset of participants that had their external and internal tibial rotation measured using the robotic knee testing (RKT) device; Table S5: The *COL12A1* rs970547 (A/G) genotype effects on the (i) passive and active genu recurvatum measurements, and (ii) anterior (Ant) and posterior (Post) tibial translation (displacement), active and maximum (Max) displacement, as well as the compliance index measured using the KT-1000 arthrometer of participants uninjured dominant and nondominant legs; Table S6: The *TNC* rs1061494 (T/C) genotype effects on the (i) passive and active genu recurvatum measurements, and (ii) anterior (Ant) and posterior (Post) tibial translation (displacement), active and maximum (Max) displacement, as well as compliance index, measured using the KT-1000 arthrometer of participants uninjured dominant and non-dominant legs; Table S7: The *TNC* rs1138545 (C/T) genotype effects on the (i) passive and active genu recurvatum measurements, (ii) anterior (Ant) and posterior (Post) tibial translation (displacement), active and maximum (Max) displacement, as well as compliance index measured using the KT-1000 arthrometer, and (iii) external and internal tibial rotation, as well as slack measured using the robotic knee testing (RKT) device of participants uninjured dominant and non-dominant legs; Table S8: The *TNC* rs2104772 (T/A) genotype effects on the (i) passive and active genu recurvatum measurements, (ii) anterior (Ant) and posterior (Post) tibial translation (displacement), active and maximum (Max) displacement, as well as compliance index measured using the KT-1000 arthrometer, and (iii) external and internal tibial rotation, as well as slack measured using the robotic knee testing (RKT) device of participants uninjured dominant and non-dominant legs; Table S9: The *COL1A1* rs1107946 (G/T) and rs1800012 (G/T) genotype effects on the participants (i) general characteristics, (ii) Beighton score and sit and reach measurements, and (iii) external and internal tibial rotation, as well as slack, measured using the robotic knee testing (RKT) device of the uninjured dominant leg; Table S10: The *COL1A1* rs1107946 (G/T) and rs1800012 (G/T) genotype–genotype effects on the participants (i) general characteristics, (ii) Beighton score and sit and reach measurements, and (iii) external and internal tibial rotation, as well as slack, measured using the robotic knee testing (RKT) device of the uninjured dominant leg; Table S11: Spearman’s correlation (r) and *p*-value (*p*) between the participants age, height, weight, and body mass index (BMI), as well as differences in sex, with external (Ext) and internal (Int) tibial rotation, as well as slack of the uninjured dominant leg; Table S12: The *COL12A1* rs970547 (A/G) genotype effects on the absolute change in the calculated length of the anterior bundle of the ACL (aACL), posterior bundle of the ACL (pACL), anterior bundle of the PCL (aPCL), posterior bundle of the PCL (pPCL), and the LCL during internal to external tibial rotation of the uninjured dominant and non-dominant legs; Table S13: The *TNC* rs1061494 TT versus the combined TC and CC genotype effects on the absolute change in the calculated length of the anterior bundle of the ACL (aACL), posterior bundle of the ACL (pACL), anterior bundle of the PCL (aPCL), posterior bundle of the PCL (pPCL), anterior bundle of the superficial layer of the MCL (aMCL), inferior bundle of the superficial layer of the MCL (iMCL), posterior bundle of the superficial layer of the MCL (pMCL), anterior bundle of the deep layer of the MCL (aDMCL), posterior bundle of the deep layer of the MCL (pDMCL) and the LCL during internal to external tibial rotation of the uninjured dominant and non-dominant legs; Table S14: The *TNC* rs1061494 (T/C) genotype effects on the absolute change in the calculated length of the anterior bundle of the anterior bundle of the PCL (aPCL), posterior bundle of the PCL (pPCL), anterior bundle of the superficial layer of the MCL (aMCL), inferior bundle of the superficial layer of the MCL (iMCL), posterior bundle of the superficial layer of the MCL (pMCL), anterior bundle of the deep layer of the MCL (aDMCL), posterior bundle of the deep layer of the MCL (pDMCL) and the LCL during internal to external tibial rotation of the uninjured dominant and non-dominant legs; Table S15: The *TNC* rs2104772 (T/A) genotype effects on the absolute change in the calculated length of the anterior bundle of the ACL (aACL), posterior bundle of the ACL (pACL), anterior bundle of the PCL (aPCL), posterior bundle of the PCL (pPCL), anterior bundle of the superficial layer of the MCL (aMCL), inferior bundle of the superficial layer of the MCL (iMCL), posterior bundle of the superficial layer of the MCL (pMCL), anterior bundle of the deep layer of the MCL (aDMCL), posterior bundle of the deep layer of the MCL (pDMCL) and the LCL during internal to external tibial rotation of the uninjured dominant and non-dominant legs; Table S16: The TNC rs1138545 (C/T) genotype effects on the absolute change in the calculated length of the anterior bundle of the ACL (aACL), posterior bundle of the ACL (pACL), anterior bundle of the PCL (aPCL), posterior bundle of the PCL (pPCL), anterior bundle of the superficial layer of the MCL (aMCL), inferior bundle of the superficial layer of the MCL (iMCL), posterior bundle of the superficial layer of the MCL (pMCL), anterior bundle of the deep layer of the MCL (aDMCL), posterior bundle of the deep layer of the MCL (pDMCL) and the LCL; Table S17: The combined *COL1A1* rs1107946 (G/T) and rs18000012 (G/T) genotype effects on the absolute change in the calculated length of the anterior bundle of the ACL (aACL), posterior bundle of the ACL (pACL), anterior bundle of the PCL (aPCL), posterior bundle of the PCL (pPCL), anterior bundle of the superficial layer of the MCL (aMCL), inferior bundle of the superficial layer of the MCL (iMCL), posterior bundle of the superficial layer of the MCL (pMCL), anterior bundle of the deep layer of the MCL (aDMCL), posterior bundle of the deep layer of the MCL (pDMCL) and the LCL during internal to external tibial rotation of the uninjured dominant and non-dominant legs; Figure S1: The *COL12A1* rs970547 (A/G) genotype effects on the five medial collateral ligament (MCL) bundle (anterior deep layer, posterior deep layer, anterior, posterior, and intermediate) lengths within the dominant leg from maximum (Max) external (ER) at to maximum internal (IR) rotation; Figure S2: The *COL12A1* rs970547 (A/G) genotype effects on the five medial collateral ligament (MCL) bundle (anterior deep layer, posterior deep layer, anterior, posterior, and intermediate) lengths within the nondominant leg from maximum (Max) external (ER) at to maximum internal (IR) rotation; Figure S3: The *TNC* rs1061494 (T/C) genotype effects on the five medial collateral ligament (MCL) bundle (anterior deep layer, posterior deep layer, anterior, posterior, and intermediate) lengths within the dominant leg from maximum (Max) external (ER) at to maximum internal (IR) rotation; Figure S4: The *TNC* rs1061494 (T/C) genotype effects on the five medial collateral ligament (MCL) bundle (anterior deep layer, posterior deep layer, anterior, posterior, and intermediate) lengths within the nondominant leg from maximum (Max) external (ER) at to maximum internal (IR) rotation. Figure S5: The *TNC* rs1061494 (T/C) genotype effects on the anterior and posterior ACL bundles lengths within the dominant leg from maximum (Max) external (ER) to maximum internal (IR) rotation.
