# Supplementary material for: The Association of Genetic Variants Within the Type XII Collagen and Tenascin C Genes with Knee Joint Laxity Measurements
Source: Genes (Basel). 2025 Jan 27;16(2):164. doi: 10.3390/genes16020164 (PMC11855217; doi:10.3390/genes16020164)
Supplement: Supplementary file 1 [file genes-16-00164-s001.zip › genes-3377052-supplementary.pdf]

SUPPLEMENTARY MATERIAL

Table S1: The *COL12A1* rs970547 (A/G) genotype effects on all the participants general characteristics, Beighton score and sit and reach measurements, as well as the subset of participants that had their external and internal tibial rotation measured using the Robotic Knee Testing (RKT) device.

COL12A1 **rs970547**	**All Particiopants**			**External and Internal Rotation**		
	**AA**	**AG + GG** [a]	**P-value**	**AA**	**AG + GG** [b]	**P-value**
	n = 71	n = 56		n = 40	n = 38	
**Sex** (% male)	57.7 (41)	57.1 (32)	1.000	65.0 (26)	57.9 (22)	0.642
**Age** (years)	26.0 (24.0; 32.0)	27.0 (24.3; 32.3)	0.280	25.5 (23.0; 28.8)	28.0 (25.0; 30.8)	0.067
**Height** (cm)	174.8 ± 10.2	174.0 ± 9.4	0.674	175.7 ± 9.7	174.5 ± 9.8	0.585
**Weight** (kg)	74.4 ± 13.3	71.1 ± 12.4	0.149	75.0 (67.7; 87.4)	74.2 (60.5; 79.3)	0.095
**BMI** (kg/m^2)	23.8 (22.1; 25.7)	23.5 (21.1; 25.7)	0.204	24.1 (22.7; 26.4)	23.7 (21.2; 25.5)	0.106
**Flexibility Training** (% Yes)	59.2 (42)	50.0 (28)	0.370	60.0 (24)	50.0 (19)	0.495
**Beighton Score**	1.0 (0.0; 3.0)	1.5 (0.0; 5.0)	0.391	2.0 (0.0; 3.0)	1.5 (0.0; 5.0)	0.511
**Sit and Reach** (cm)	43.9 ± 10.6 [c]	41.2 ± 10.4 [d]	0.161	46.1 ± 9.6 [e]	41.1 ± 10.5	**0.034**

Sex and flexibility training are reported as relative percentages with number of males and participants who do flexibilibity taining, respectively, in paraenthesis. The remaining continuous variables are reported as average ± standard deviation or median (IQR) for parametric and non-parametric data respectively

Unable to genotype 1 participant for rs970547

Significant differences are indicated in bold

BMI, body mass index

The rare GG ([a] n = 7, [b] n = 6) genotype and the GA genotype were combined for the analysis

[c] n = 68, [d] n = 55, [e] n = 37

Table S2: The *TNC* rs1061494 (T/C) genotype effects on all the participants general characteristics, Beighton score and sit and reach measurements, as well as the subset of participants that had their external and internal tibial rotation measured using the Robotic Knee Testing (RKT) device.

TNC rs1061494	All Particiopants				External and Internal Rotation			
	TT	TC	CC	P-Value	TT	TC	CC	P-Value
	n = 38	n = 62	n = 28		n = 25	n = 35	n = 18	
**Sex** (% male)	47.4 (18)	59.7 (37)	67.9 (19)	0.229	60.0 (15)	54.3 (19)	77.8 (14)	1.000 [a]
**Age** (years)	25.0 (23.0; 33.0)	27.0 (24.8; 30.0)	30.0 (24.5; 34.5)	0.176	25.0 (23.0; 28.5)	27.0 (25.0; 32.3)	28.5 (25.5; 32.3)	0.091
**Height** (cm)	173.0 ± 9.8	175.9 ± 9.8	173.8 ± 9.8	0.304	174.2 ± 10.6	174.6 ± 9.7	177.2 ± 8.6	0.569
**Weight** (kg)	71.9 (60.2; 80.7)	74.9 (62.1; 84.5)	73.5 (62.1; 78.3)	0.474	74.3 ± 12.6	73.2 ± 13.1	76.0 ± 11.7	0.293
**BMI** (kg/m^2)	23.8 (22.1; 25.2)	23.8 (21.2; 26.1)	23.4 (21.5; 25.8)	0.949	24.0 (22.8; 25.7)	23.8 (21.2; 25.5)	23.9 (21.6; 25.9)	0.662
**Flexibility Training** (% Yes)	47.4 (18)	56.5 (35)	60.7 (17)	0.520	48.0 (12)	57.1 (20)	61.1 (11)	0.660
**Beighton Score**	1.0 (0.0; 3.3)	2.0 (0.0; 4.0)	1.0 (0.0; 4.8)	0.486	1.0 (0.0; 4.0)	3.0 (1.0; 4.0)	1.0 (0.0; 4.0)	0.379
**Sit and Reach** (cm)	41.9 ± 9.8	42.7 ± 11.8 [b]	42.8 ± 10.1 [c]	0.929	41.0 (35.3; 47.8)	45.4 (33.6; 54.1) [d]	44.0 (41.3; 48.5) [e]	0.330

Sex and flexibility training are reported as relative percentages with number of males and participants who do flexibilibity taining, respectively, in paraenthesis. The remaining continuous variables are reported as average ± standard deviation or median (IQR) for parametric and non-parametric data respectively

BMI, body mass index

[a] The TC and CC genotypes were combined for the analysis because there were only 4 females in the CC genotype group

[b] n = 60, [c] n = 26, [d] n = 34, [e] n = 16

Table S3: The *TNC* rs1138545 (C/T) genotype effects on all the participants general characteristics, Beighton score and sit and reach measurements, as well as the subset of participants that had their external and internal tibial rotation measured using the Robotic Knee Testing (RKT) device.

***TNC* rs1138545**	**All Particiopants**			**External and Internal Rotation**		
	**CC**	**CT + TT [a]**	**P-value**	**CC**	**CT + TT [b]**	**P-value**
	n = 95	n = 31		n = 57	n = 20	
**Sex** (% male)	62.1 (59)	45.2 (14)	0.142	63.2 (36)	60.0 (12)	0.796
**Age** (years)	26.0 (24.0; 32.0)	28.0 (25.0; 33.0)	0.182	26.0 (24.0; 30.0)	29.0 (25.3; 33.0)	0.089
**Height** (cm)	176.0 (167.0; 182.4)	171.2 (164.4; 181.2)	0.135	175.9 ± 9.7	173.5 ± 9.5	0.338
**Weight** (kg)	74.4 (62.4; 81.5)	71.0 (60.2; 80.9)	0.275	74.4 ± 12.1	74.4 ± 13.8	0.999
**BMI** (kg/m^2)	24.0 ± 3.0	23.8 ± 3.2	0.827	24.0 ± 2.8	24.6 ± 3.4	0.410
**Flexibility Training** (% Yes)	53.7 (95)	58.1 (18)	0.685	54.4 (31)	55.0 (11)	1.000
**Beighton Score**	1.0 (0.0; 4.0)	2.0 (1.0; 5.0)	0.155	1.0 (0.0; 4.0)	2.0 (1.0; 4.8)	0.296
**Sit and Reach** (cm)	42.6 ± 10.6 [c]	43.6 ± 10.2	0.633	44.4 ± 9.6 [d]	42.2 ± 11.6	0.405

Sex and flexibility training are reported as relative percentages. The remaining continuous variables are reported as average ± standard deviation or median (IQR) for parametric and non-parametric data respectively

Unable to genotype 2 participants for rs1138545

BMI, body mass index

The rare TT ([a] n = 4, [b] n = 1) genotype and the CT genotype were combined for the analysis

[c] n = 91, [d] n = 54

Table S4: The *TNC* rs2104772 (T/A) genotype effects on all the participants general characteristics, Beighton score and sit and reach measurements, as well as the subset of participants that had their external and internal tibial rotation measured using the Robotic Knee Testing (RKT) device.

TNC **rs2104772**	**All Particiopants**				**External and Internal Rotation**			
	**TT**	**TA**	**AA**	**P-Value**	**TT**	**TA**	**AA**	**P-Value**
	n = 50	n = 62	n = 15		n = 30	n = 38	n = 10	
**Sex** (% male)	64.0 (32)	53.2 (33)	60.0 (9)	0.511	63.3 (19)	57.9 (22)	70.0 (7)	0.816 [a]
**Age** (years)	26.0 (23.0; 30.0)	27.0 (24.0; 34.0)	27.0 (26.0; 32.0)	0.125	26.0 (23.0; 29.3)	27.5 (23.8; 33.3)	26.0 (25.0; 29.3)	0.430
**Height** (cm)	175.4 ± 9.5	173.4 ± 9.9	176.2 ± 10.8	0.803	175.1 (171.8; 181.0)	174.3 (164.4; 182.6)	183.0 (173.7; 186.2)	0.203
**Weight** (kg)	75.8 (62.4; 86.6)	73.1 (60.5; 80.6)	76.5 (61.8; 88.0)	0.301	74.2 ± 13.3	72.5 ± 11.5	80.8 ± 12.7	0.177
**BMI** (kg/m^2)	23.9 (21.1; 26.1)	23.6 (21.5; 25.2)	23.8 (21.9; 25.8)	0.604	24.0 ± 2.8	24.0 ± 2.8	25.0 ± 3.8	0.577
**Flexibility Training** (% Yes)	54.0 (27)	54.8 (34)	60.0 (9)	0.918	53.3 (16)	55.3 (21)	60.0 (6)	0.819 [a]
**Beighton Score**	2.0 (0.0; 4.0)	1.0 (0.0; 4.0)	1.0 (1.0; 4.0)	0.708	2.0 (0.0; 4.0)	1.0 (0.0; 3.3)	1.5 (0.8; 4.3)	0.623
**Sit and Reach** (cm)	41.4 ± 9.6 [b]	42.3 ± 12.1 [c]	46.6 ± 8.7	0.271	43.0 ± 9.0 [d]	43.3 ± 12.3 [e]	46.1 ± 5.2	0.704

Sex and flexibility training are reported as relative percentages. The remaining continuous variables are reported as average ± standard deviation or median (IQR) for parametric and non-parametric data respectively

Unable to genotype 1 participant for rs2104772

BMI, body mass index

[a] The TA and AA genotypes were combined for the analysis because there were only 3 females and 4 particpants who did not do any flexibility training in the AA genotype group

[b] n = 48, [c] n = 60, [d] n = 29, [e] n = 36

Table S5: The *COL12A1* rs970547 (A/G) genotype effects on the (i) passive and active genu recurvatum measurements, and (ii) anterior (Ant) and posterior (Post) tibial translation (displacement), active and maximum (Max) displacement, as well as compliance index measured using the KT-1000 arthrometer of participants unijured dominant and non-dominant legs.

COL12A1 rs970547	Dominant Leg			Non-Dominant Leg		
	**AA**	**AG + GG**	**P-value**	**AA**	**AG + GG**	**P-value**
**Genu Recurvatum**	n = 64	n = 49 [c]		n = 68	n = 52 [c]	
▪ **Passive** (°)	177.7 ± 5.3	177.0 ± 6.5	0.533	177.3 ± 5.0	176.8 ± 6.6	0.670
▪ **Active** (°)	175.8 ± 5.2	174.8 ± 6.4	0.457	175.0 ± 5.0	174.9 ± 6.2	0.933
**Ant-Post Translation**	n = 64	n = 49 [c]		n = 68	n = 52 [c]	
▪ **133 N Ant Translation** (mm)	6.0 (5.0; 8.5)	6.5 (5.0; 8.3)	0.927	7.4 ± 2.2	7.0 ± 2.7	0.387
▪ **133 N Post Translation** (mm)	4.5 (3.0; 6.0) [d]	4.0 (3.0; 5.3)	0.251	4.0 (3.0; 5.5) [e]	3.8 (3.0; 5.0)	0.543
▪ **Compliance Index** (mm) [b]	4.7 ± 1.9	4.6 ± 1.9	0.794	4.9 ± 1.9	4.6 ± 2.1	0.350
▪ **Active Displacement** (mm)	4.0 (2.5; 6.0)	4.0 (2.0; 6.0)	0.753	4.9 ± 2.5	4.9 ± 2.9 [f]	0.940
▪ **Max Displacement** (mm)	7.0 (5.1; 8.5)	7.0 (5.5; 9.3)	0.911	7.0 (5.0; 10.0)	7.5 (6.0; 9.9)	0.405

Values are expressed as either average ± standard deviation or median (IQR) for parametric and non-parametric data respectively

Unable to genotype 1 participant for rs970547

[a] Lower degree values for genu recurvatum indicates a greater amount of hyperextension

[b] Compliance index = 133 N - 67 N Anterior Translation

The rare GG ([c] n = 7) genotype and the GA genotype were combined for the analysis

[d] n = 63, [e] n = 67, [f] n = 51

Table S6: The *TNC* rs1061494 (T/C) genotype effects on the (i) passive and active genu recurvatum measurements, and (ii) anterior (Ant) and posterior (Post) tibial translation (displacement), active and maximum (Max) displacement, as well as compliance index, measured using the KT-1000 arthrometer of participants unijured dominant and non-dominant legs.

***TNC* rs1061494**	**Dominant Leg**				**Non-Dominant Leg**			
	**TT**	**TC**	**CC**	**P-Value**	**TT**	**TC**	**CC**	**P-Value**
**Genu Recurvatum** [a]	n = 37	n = 55	n = 22		n = 37	n = 58	n = 25	
▪ **Passive** (°)	178.4 ± 5.4	176.3 ± 6.5	178.7 ± 4.2	0.249	178.4 ± 5.9	176.6 ± 6.0	176.4 ± 4.6	0.254
▪ **Active** (°)	176.4 ± 5.3	174.0 ± 6.1	176.6 ± 4.8	0.066	175.7 ± 5.7	174.6 ± 5.6	174.6 ± 5.0	0.576
**Ant-Post Translation**	n = 37	n = 55	n = 22		n = 37	n = 58	n = 25	
▪ **133 N Ant Translation** (mm)	6.5 (5.0; 8.3)	6.5 (5.5; 8.5)	6.0 (4.9; 8.1)	0.814	7.2 ± 2.3	7.1 ± 2.4	7.4 ± 2.9	0.091
▪ **133 N Post Translation** (mm)	4.0 (3.5; 6.0)	4.0 (3.0; 6.0)	4.0 (3.0; 5.0) [c]	0.835	3.5 (3.0; 5.0)	4.3 (3.0; 6.0)	4.0 (3.0; 5.0) [d]	0.308
▪ **Compliance Index** (mm) [b]	4.6 ± 1.8	4.9 ± 2.0	4.4 ± 1.7	0.532	4.5 (3.5; 6.5)	4.3 (3.0; 6.0)	4.5 (3.0; 7.0)	0.478
▪ **Active Displacement** (mm)	4.0 (2.0; 5.8)	4.0 (3.0; 6.0)	3.3 (1.0; 6.0)	0.270	4.5 (2.8; 7.0)	5.0 (3.0; 7.0) [e]	5.0 (1.8; 7.0)	0.886
▪ **Max Displacement** (mm)	7.0 (5.3; 8.5)	7.0 (6.0; 8.5)	7.3 (4.5; 10.0)	0.637	7.0 (5.0; 10.0)	7.0 (6.0; 10.3)	7.0 (5.0; 10.0)	0.497

Values are expressed as either average ± standard deviation or median (IQR) for parametric and non-parametric data respectively

[a] Lower degree values for genu recurvatum indicates a greater amount of hyperextension

[b] Compliance index = 133 N - 67 N Anterior Translation

[c] n = 21, [d] n = 24, [e] n = 57

Table S7: The *TNC* rs1138545 (C/T) genotype effects on the (i) passive and active genu recurvatum measurements, (ii) anterior (Ant) and posterior (Post) tibial translation (displacement), active and maximum (Max) displacement, as well as compliance index measured using the KT-1000 arthrometer, and (iii) external and internal tibial rotation, as well as slack measured using the Robotic Knee Testing (RKT) device of participants uninjured dominant and non-dominant legs.

TNC rs1138545	Dominant Leg			Non-Dominant Leg		
	CC	CT + TT	P-value	CC	CT + TT	P-value
**Genu Recurvatum** [a]	n = 86	n = 26 [c]		n = 87	n = 31 [c]	
▪ **Passive** (°)	179.0 (174.0; 182.0)	177.3 (172.0; 182.4)	0.596	177.2 ± 5.8	177.1 ± 5.7	0.968
▪ **Active** (°)	175.4 ± 5.5	175.6 ± 6.1	0.897	175.2 ± 5.5	174.6 ± 5.5	0.630
**Ant-Post Translation**	n = 86	n = 26 [d]		n = 87	n = 31 [d]	
▪ **133 N Ant Translation** (mm)	6.0 (5.0; 8.5)	7.0 (5.9; 7.6)	0.608	7.1 ± 2.4	7.5 ± 2.8	0.433
▪ **133 N Post Translation** (mm)	4.0 (3.0; 5.8) [f]	4.0 (3.0; 6.0)	0.406	4.0 (3.0; 5.0) [g]	5.0 (3.0; 6.0)	0.125
▪ **Compliance Index** (mm) [b]	4.7 ± 1.9	4.7 ± 1.7	0.896	4.7 ± 1.9	5.1 ± 2.2	0.334
▪ **Active Displacement** (mm)	4.0 (2.0; 6.0)	3.8 (3.0; 6.0)	0.666	4.9 ± 2.6	5.0 ± 2.9 [h]	0.878
▪ **Max Displacement** (mm)	7.0 (5.4; 9.0)	7.0 (5.5; 8.1)	0.707	7.5 (5.0; 10.0)	6.5 (6.0; 10.0)	0.752
**External-internal Rotation**	n = 49	n = 16 [e]		n = 54	n = 20 [e]	
▪ **External Rotation** (°)	5.4 ± 1.4	5.5 ± 1.5	0.746	5.4 ± 1.2	5.4 ± 1.3	0.965
▪ **Internal Rotation** (°)	5.7 ± 1.0	5.6 ± 1.5	0.665	5.5 (5.0; 6.3)	5.6 (5.1; 6.4)	0.614
▪ **Slack** (°) [c]	17.5 ± 3.4	17.6 ± 4.4	0.942	17.3 ± 3.0	17.8 ± 4.1	0.603

Values are expressed as either average ± standard deviation or median (IQR) for parametric and non-parametric data respectively

Unable to genotype 2 participants for rs1138545

[a] Lower degree values for genu recurvatum indicates a greater amount of hyperextension

[b] Compliance index = 133 N - 67 N Anterior Translation

[c] Slack is defined as the amount of tibial rotation which occurred between the two turning points of the load deformation curve in the area of play between flanking external and internal rotation regions

The rare TT ([d] n = 4, [e] n = 1) genotype and the CT genotype were combined for the analysis

[f] n = 85, [g] n = 86, [h] n = 30

Table S8: The *TNC* rs2104772 (T/A) genotype effects on the (i) passive and active genu recurvatum measurements, (ii) anterior (Ant) and posterior (Post) tibial translation (displacement), active and maximum (Max) displacement, as well as compliance index measured using the KT-1000 arthrometer, and (iii) external and internal tibial rotation, as well as slack measured using the Robotic Knee Testing (RKT) device of participants uninjured dominant and non-dominant legs.

TNC rs2104772	Dominant Leg				Non-Dominant Leg			
	TT	TA	AA	P-Value	TT	TA	AA	P-Value
**Genu Recurvatum** [a]	n = 43	n = 57	n = 13		n = 48	n = 57	n = 14	
▪ **Passive** (°)	180.0 (174.0; 182.0)	176.0 (172.0; 180.8)	178.0 (174.3; 181.3)	0.195	178.0 (174.0; 181.9)	176.5 (173.0; 181.3)	176.0 (174.9; 179.1)	0.795
▪ **Active** (°)	176.3 ± 5.9	174.6 ± 5.4	174.6 ± 6.5	0.315	175.5 ± 5.8	175.0 ± 5.1	173.2 ± 6.1	0.405
**Ant-Post Translation**	n = 43	n = 57	n = 13		n = 48	n = 57	n = 14	
▪ **133 N Ant Translation** (mm)	6.0 (5.0; 8.0)	6.5 (4.8; 8.3)	7.0 (5.0; 8.8)	0.695	7.0 (6.0; 9.5)	7.0 (5.5; 9.0)	7.0 (6.0; 7.9)	0.700
▪ **133 N Post Translation** (mm)	4.0 (3.0; 5.1) [d]	4.0 (3.0; 6.0)	5.0 (3.3; 7.0)	0.315	4.0 (2.5; 5.0) [e]	4.0 (3.3; 5.5)	3.8 (2.5; 5.1)	0.389
▪ **Compliance Index** (mm) [b]	4.6 ± 2.1	4.7 ± 1.8	4.8 ± 1.5	0.949	4.9 ± 2.1	4.8 ± 1.9	4.2 ± 1.7	0.578
▪ **Active Displacement** (mm)	4.0 (2.0; 6.0)	4.0 (3.0; 6.0)	5.0 (2.8; 7.0)	0.521	5.0 (2.5; 7.0) [e]	4.5 (3.0; 6.3)	5.3 (3.0; 8.1)	0.412
▪ **Max Displacement** (mm)	7.0 (5.0; 8.0)	7.0 (6.0; 8.8)	7.0 (4.3; 9.0)	0.749	7.3 (5.1; 10.5)	7.0 (6.0; 9.0)	7.0 (5.0; 10.3)	0.983
**External-internal Rotation**	n = 24	n = 34	n = 8		n = 30	n = 36	n = 10	
▪ **External Rotation** (°)	5.5 ± 1.5	5.3 ± 1.4	5.5 ± 1.2	0.893	5.4 (4.8; 6.2) [f]	5.4 (4.6; 6.4)	5.0 (4.0; 5.8)	0.415
▪ **Internal Rotation** (°)	6.1 (5.2; 6.6)	5.7 (4.7; 6.0)	6.0 (5.5; 7.2)	0.191	5.8 (5.2; 6.6)	5.5 (5.1; 6.3)	5.4 (4.5; 6.0)	0.250
▪ **Slack** (°) [c]	18.1 ± 3.7	17.1 ± 3.7	17.2 ± 3.2	0.584	17.5 (16.0; 20.0)	17.1 (14.5; 20.0)	15.8 (14.9; 18.9)	0.230

Values are expressed as either average ± standard deviation or median (IQR) for parametric and non-parametric data respectively

Unable to genotype 1 participant for rs2104772

[a] Lower degree values for genu recurvatum indicates a greater amount of hyperextension

[b] Compliance index = 133 N - 67 N Anterior Translation

[c] Slack is defined as the amount of tibial rotation which occurred between the two turning points of the load deformation curve in the area of play between flanking external and internal rotation regions

[d] n = 42, [e] n = 47, [f] n = 29

Table S9: The *COL1A1* rs1107946 (G/T) and rs1800012 (G/T) genotype effects on the participants (i) general characteristics, (ii) Beighton score and sit and reach measurements, and (iii) external and internal tibial rotation, as well as slack, measured using the Robotic Knee Testing (RKT) device of the uninjured dominant leg.

	***COL1A1* rs1107946 [a]**			***COL1A1* rs1800012**		
	**GG**	**GT**	**P-value**	**GG**	**GT + TT [b]**	**P-value**
	n = 45	n = 21		n = 43	n = 23	
**Sex** (% male)	62.2 (28)	52.4 (11)	0.592	58.1 (25)	60.9 (14)	1.000
**Age** (years)	26.0 (24.5; 29.5)	26.0 (23.0; 30.0)	0.698	26.0 (24.0; 29.0)	27.0 (24.0; 35.0)	0.096
**Height** (cm)	175.9 ± 9.3	172.1 ± 10.7	0.139	174.3 ± 10.4	175.4 ± 8.9	0.659
**Weight** (kg)	75.3 ± 12.7	71.5 ± 11.8	0.245	72.8 ± 12.4	76.6 ± 12.4	0.235
**BMI** (kg/m^2)	24.0 (22.6; 25.9)	23.7 (22.5; 25.6)	0.591	23.8 ± 2.2	24.9 ± 3.9	0.143
**Flexibility Training** (% Yes)	62.2 (28)	47.6 (10)	0.295	62.8 (27)	47.8 (11)	0.300
**Beighton Score**	1.0 (0.0; 4.0)	3.0 (0.0; 5.0)	0.892	2.0 (0.0; 4.0)	1.0 (0.0; 3.0)	0.246
**Sit and Reach** (cm)	44.6 ± 8.9 [c]	41.4 ± 12.8 [d]	0.260	44.5 ± 10.3 [e]	41.7 ± 10.5 [f]	0.313
**External Tibial Rotation** (°)	5.3 ± 1.6	5.4 ± 1.3	0.842	5.5 ± 1.6	5.3 ± 1.1	0.576
**Internal Tibial Rotation** (°)	5.7 (5.0; 6.2)	5.5 (4.3; 6.0)	0.189	5.8 ± 1.1	5.4 ± 1.1	0.105
**Slack** (°)	16.9 (15.0; 19.4)	16.8 (13.9; 18.9)	0.497	17.9 ± 3.9	16.7 ± 3.0	0.199

Sex and flexibility training are reported as relative percentages, while the remaining continuous variables are reported as average ± standard deviation or median (IQR) for parametric and non-parametric data respectively

BMI, body mass index

[a] None of the participants had the rare rs1107946 TT genotype

[b] The rare TT (n = 1) and GT genotypes of rs1800012 were combined for the analysis

[c] n = 43, [d] n = 20, [e] n = 41, [f] n = 23

Table S10: The *COL1A1* rs1107946 (G/T) and rs1800012 (G/T) genotype-genotype effects on the participants (i) general characteristics, (ii) Beighton score and sit and reach measurements, and (iii) external and internal tibial rotation, as well as slack, measured using the Robotic Knee Testing (RKT) device of the uninjured dominant leg.

COL1A1	**rs1107946 GG and rs1800012 GG**	**Remaining Genotype Combinations**	**P-value**
	n = 30	n = 36	
**Sex** (% male)	63.3 (19)	55.6 (20)	0.618
**Age** (years)	26.0 (24.8; 29.3)	26.5 (23.0; 30.0)	0.707
**Height** (cm)	175.3 ± 9.9	174.2 ± 9.9	0.682
**Weight** (kg)	74.3 ± 11.9	73.9 ± 13.1	0.881
**BMI** (kg/m^2)	24.1 ± 2.2	24.3 ± 3.5	0.788
**Flexibility Training** (% Yes)	63.3 (19)	52.8 (19)	0.458
**Beighton Score**	2.0 (0.0; 4.0)	1.0 (0.0; 4.0)	0.627
**Sit and Reach** (cm)	45.0 ± 9.6 [a]	42.3 ± 11.0 [b]	0.304
**External Tibial Rotation** (°)	5.3 (4.8; 6.6)	5.2 (4.5; 5.8)	0.524
**Internal Tibial Rotation** (°)	6.0 ± 1.1	5.4 ± 1.0	**0.025**
**Slack** (°)	18.2 ± 3.9	16.9 ± 3.3	0.126

Sex and flexibility training are reported as relative percentages, while the remaining continuous variables are reported as average ± standard deviation or median (IQR) for parametric and non-parametric data respectively

BMI, body mass index

Significant differences are indicated in bold

[a] n = 29; [b] n = 34

Table S11: Spearman's correlation (r) and P-value (P) between the participants age, height, weight and body mass index (BMI), as well as differences in sex, with external (Ext) and internal (Int) tibial rotation, as well as slack of the uninjured dominant leg.

	n	Age	Height	Weight	BMI	Sex
**Ext Rotattion** (°)	66	r = -0.100<br>P = 0.424	r = -0.096<br>P = 0.444	r = -0.235<br>P = 0.058	r = -0.238<br>P = 0.054	**M: 5.1 ± 1.2**<br>**F: 5.9 ± 1.6**<br>**P = 0.022**
**Int Rotation** (°)	66	r = -0.179<br>P = 0.151	r = 0.011<br>P = 0.930	**r = -0.271**<br>**P = 0.028**	**r = -0.389**<br>**P = 0.001**	M: 5.6 ± 1.0<br>F: 5.9 ± 1.3<br>P = 0.293
**Slack** (°) [a]	66	r = -0.185<br>P = 0.138	r = -0.133<br>P = 0.289	**r = -0.333**<br>**P = 0.006**	**r = -0.338**<br>**P = 0.006**	**M: 16.8 ± 3.1**<br>**F: 18.6 ± 4.1**<br>**P = 0.045**

Significant correlations and differences are in bold

The number of partipants (n) in each analysis is indicated

The male (M) and female (F) values are represented as average ± standard deviation

[a] Slack is defined as the amount of tibial rotation which occurred between the two turning points of the load deformation curve in the area of play between flanking external and internal rotation regions

Table S12: The *COL12A1* rs970547 (A/G) genotype effects on the absolute change in the calculated length of the anterior bundle of the ACL (aACL), posterior bundle of the ACL (pACL), anterior bundle of the PCL (aPCL), posterior bundle of the PCL (pPCL), and the LCL during internal to external tibial rotation of the uninjured dominant and non-dominant legs.

***COL12A1* rs970547**	**Dominant Leg**			**Non-Dominant Leg**		
	**AA**	**AG + GG**	**P-value**	**AA**	**AG + GG**	**P-value**
	n = 36	n = 30 [a]		n = 40	n = 35 [b]	
**aACL** (mm)	1.0 (0.8; 1.5)	1.4 (1.0; 1.7)	0.068	1.1 (0.8; 1.6)	1.2 (0.9; 1.5)	0.837
**pACL** (mm)	3.0 ± 1.5	3.3 ± 1.4	0.352	3.0 ± 1.3	2.9 ± 1.4	0.673
**aPCL** (mm)	2.0 (1.1; 2.8)	2.0 (1.6; 3.3)	0.954	1.5 (0.9; 2.6)	2.0 (1.3; 3.2)	0.202
**pPCL** (mm)	1.5 (0.6; 2.3)	1.4 (0.7; 2.2)	0.431	1.3 (0.8; 2.5)	1.4 (0.9; 2.1)	0.747
**LCL** (mm)	1.5 (0.9; 1.9)	1.3 (0.9; 1.9)	0.652	1.1 (0.9; 1.5)	1.2 (0.8; 1.9)	0.829

Values are expressed as average ± standard deviation or median (IQR) for parametric and non-parametric data respectively

The rare GG ([a] n = 4 and [b] n = 5) genotype and the GA genotype were combined for the analysis

Table S13: The *TNC* rs1061494 TT versus the combined TC and CC genotype effects on the absolute change in the calculated length of the anterior bundle of the ACL (aACL), posterior bundle of the ACL (pACL), anterior bundle of the PCL (aPCL), posterior bundle of the PCL (pPCL), anterior bundle of the superficial layer of the MCL (aMCL), inferior bundle of the superficial layer of the MCL (iMCL), posterior bundle of the superficial layer of the MCL (pMCL), anterior bundle of the deep layer of the MCL (aDMCL), posterior bundle of the deep layer of the MCL (pDMCL) and the LCL during internal to external tibial rotation of the uninjured dominant and non-dominant legs.

TNC rs1061494	**Dominant Leg**			**Non-Dominant Leg**		
	**TT**	**TC + CC**	**P-value**	**TT**	**TC + CC**	**P-value**
	n = 24	n = 42		n = 24	n = 51	
**aACL** (mm)	1.0 (0.8; 1.6)	1.3 (1.0; 1.6)	0.145	1.1 (0.8; 1.3)	1.3 (0.8; 1.6)	0.317
**pACL** (mm)	3.0 ± 1.6	3.2 ± 1.4	0.381	2.7 (1.7; 3.4)	2.8 (2.1; 3.7)	0.491
**aPCL** (mm)	1.9 ± 1.1	2.3 ± 1.2	0.282	1.5 (1.0; 3.0)	1.9 (1.1; 2.6)	0.987
**pPCL** (mm)	1.1 (0.6; 2.1)	1.5 (1.1; 2.2)	0.224	1.1 (0.6; 2.2)	1.4 (1.1; 2.5)	0.195
**aMCL** (mm)	3.8 ± 1.3	4.5 ± 1.4	**0.045**	3.6 ± 1.3	4.1 ± 1.6	0.142
**iMCL** (mm)	3.4 ± 1.1	4.0 ± 1.3	**0.043**	3.1 ± 1.1	3.6 ± 1.4	0.214
**pMCL** (mm)	4.1 ± 1.1	4.7 ± 1.3	0.067	3.8 ± 1.2	4.2 ± 1.6	0.284
**aDMCL** (mm)	6.0 ± 2.0	7.3 ± 2.0	**0.020**	5.5 (4.7;6.3)	5.9 (4.8; 7.9)	0.246
**pDMCL** (mm)	6.8 ± 2.0	9.1 ± 2.2	**0.023**	6.1 (5.3; 7.4)	6.8 (5.4; 8.9)	0.446
**LCL** (mm)	1.2 (0.9; 1.7)	1.6 (1.0; 2.2)	**0.049**	1.0 (0.8; 1.5)	1.3 (0.9; 1.8)	0.345

Values are expressed as average ± standard deviation or median (IQR) for parametric and non-parametric data respectively
Significant differences are in bold

Table S14: The *TNC* rs1061494 (T/C) genotype effects on the absolute change in the calculated length of the anterior bundle of the anterior bundle of the PCL (aPCL), posterior bundle of the PCL (pPCL), anterior bundle of the superficial layer of the MCL (aMCL), inferior bundle of the superficial layer of the MCL (iMCL) , posterior bundle of the superficial layer of the MCL (pMCL), anterior bundle of the deep layer of the MCL (aDMCL), posterior bundle of the deep layer of the MCL (pDMCL) and the LCL during internal to external tibial rotation of the uninjured dominant and non-dominant legs.

TNC **rs1061494**	**Dominant Leg**				**Non-Dominant Leg**			
	**TT**	**TC**	**CC**	**P-Value**	**TT**	**TC**	**CC**	**P-Value**
	n = 24	n = 28	n = 14		n = 24	n = 34	n = 17	
**aPCL** (mm)	1.9 ± 1.1	2.5 ± 1.3	1.8 ± 0.9	0.086	1.5 (1.0; 3.0)	2.1 (1.4; 3.4)	1.2 (0.9; 2.2)	0.079
**pPCL** (mm)	1.1 (0.6; 2.1)	1.5 (1.1; 2.5)	1.4 (1.0; 2.2)	0.422	1.1 (0.6; 2.2)	1.4 (1.0; 2.5)	1.4 (1.0; 2.4)	0.418
**aMCL** (mm)	3.8 ± 1.3	4.4 ± 1.6	4.8 ± 0.8	0.138	3.6 ± 1.3	3.9 ± 1.5	4.5 ± 1.7	0.138
**iMCL** (mm)	3.4 ± 1.1	3.9 ± 1.5	4.2 ± 0.8	0.111	3.1 ± 1.1	3.4 ± 1.4	3.8 ± 1.6	0.314
**pMCL** (mm)	4.1 ± 1.1	4.6 ± 1.5	4.8 ± 0.8	0.148	3.8 ± 1.2	4.1 ± 1.6	4.5 ± 1.6	0.390
**aDMCL** (mm)	6.0 ± 2.0	7.2 ± 2.4	7.4 ± 1.1	0.065	5.7 ± 1.6	6.3 ± 1.9	6.5 ± 2.5	0.348
**pDMCL** (mm)	6.8 ± 2.0	8.2 ± 2.6	8.0 ± 1.3	0.601	6.5 ± 1.7	7.1 ± 2.1	6.9 ± 2.5	0.551
**LCL** (mm)	1.2 (0.9; 1.7)	1.7 (1.1; 2.4)	1.5 (1.0; 2.1)	0.086	1.0 (0.8; 1.5)	1.3 (0.8; 2.0)	1.2 (0.9; 1.4)	0.464

Values are expressed as either average ± standard deviation or median (IQR) for parametric and non-parametric data respectively

Table S15: The *TNC* rs2104772 (T/A) genotype effects on the absolute change in the calculated length of the anterior bundle of the ACL (aACL), posterior bundle of the ACL (pACL), anterior bundle of the PCL (aPCL), posterior bundle of the PCL (pPCL), anterior bundle of the superficial layer of the MCL (aMCL), inferior bundle of the superficial layer of the MCL (iMCL), posterior bundle of the superficial layer of the MCL (pMCL), anterior bundle of the deep layer of the MCL (aDMCL), posterior bundle of the deep layer of the MCL (pDMCL) and the LCL during internal to external tibial rotation of the uninjured dominant and non-dominant legs.

***TNC* rs2104772**	**Dominant Leg**				**Non-Dominant Leg**			
	**TT**	**TA**	**AA**	**P-Value**	**TT**	**TA**	**AA**	**P-Value**
	n = 24	n = 34	n = 8		n = 29	n = 36	n = 10	
**aACL** (mm)	1.5 (1.0; 1.8)	1.1 (0.8; 1.4)	1.0 (1.0; 1.4)	0.089	1.2 (0.8; 1.4)	1.2 (0.8; 1.7)	1.3 (0.8; 1.4)	0.878
**pACL** (mm)	3.6 ± 1.5	2.9 ± 1.4	2.7 ± 1.1	0.088	3.2 ± 1.4	2.8 ± 1.3	3.1 ± 0.9	0.751
**aPCL** (mm)	2.0 ± 1.2	2.2 ± 1.2	2.2 ± 1.2	0.757	1.6 (1.1; 2.6)	2.2 (1.3; 3.0)	1.4 (0.8; 2.2)	0.288
**pPCL** (mm)	1.5 (0.7; 2.2)	1.4 (1.0; 2.0)	1.7 (0.4; 2.5)	0.976	1.5 (1.0; 2.3)	1.3 (0.7; 2.5)	1.2 (0.8; 1.8)	0.613
**aMCL** (mm)	4.8 ± 1.2	3.9 ± 1.4	4.3 ± 1.5	0.076	4.2 (3.3; 4.9)	3.5 (3.0; 4.4)	3.1 (2.4; 5.3)	0.140
**iMCL** (mm)	4.2 ± 1.1	3.5 ± 1.2	4.0 ± 1.2	0.087	3.7 ± 1.2	3.3 ± 1.4	3.0 ± 1.4	0.320
**pMCL** (mm)	4.8 ± 1.2	4.1 ± 1.3	4.6 ± 1.2	0.092	4.4 ± 1.3	4.0 ± 1.6	3.6 ± 1.4	0.296
**aDMCL** (mm)	7.5 (5.9; 8.9)	6.4 (4.7; 7.5)	7.1 (5.2; 8.4)	0.112	6.1 (5.0; 7.5)	5.6 (4.7; 7.4)	4.7 (3.4; 7.7)	0.212
**pDMCL** (mm)	8.1 ± 2.4	7.1 ± 8.4	8.0 ± 1.8	0.285	6.3 (5.6; 8.0)	6.5 (5.3; 8.8)	5.3 (4.4; 8.0)	0.175
**LCL** (mm)	1.3 (0.9; 1.8)	1.4 (1.0; 2.1)	1.7 (1.5; 2.1)	0.158	1.2 (0.9; 1.7)	1.2 (0.8; 1.8)	0.9 (0.8; 1.3)	0.448

Values are expressed as either average ± standard deviation or median (IQR) for parametric and non-parametric data respectively

Table S16: The *TNC* rs1138545 (C/T) genotype effects on the absolute change in the calculated length of the anterior bundle of the ACL (aACL), posterior bundle of the ACL (pACL), anterior bundle of the PCL (aPCL), posterior bundle of the PCL (pPCL), anterior bundle of the superficial layer of the MCL (aMCL), inferior bundle of the superficial layer of the MCL (iMCL), posterior bundle of the superficial layer of the MCL (pMCL), anterior bundle of the deep layer of the MCL (aDMCL), posterior bundle of the deep layer of the MCL (pDMCL) and the LCL during internal to external tibial rotation of the uninjured dominant and non-dominant legs.

TNC rs1138545	**Dominant Leg**			**Non-Dominant Leg**		
	**CC**	**CT + TT**	**P-value**	**CC**	**CT + TT**	**P-value**
	n = 49	n = 16		n = 54	n = 20	
**aACL** (mm)	1.1 (1.0; 1.6)	1.0 (0.8; 1.4)	0.235	1.1 (0.8; 1.5)	1.3 (0.8; 1.6)	0.795
**pACL** (mm)	3.3 ± 1.5	2.6 ± 1.4	0.685	3.0 ± 1.4	2.9 ± 1.1	0.685
**aPCL** (mm)	2.0 (0.9; 2.8)	2.3 (1.7; 3.7)	0.060	1.6 (1.1; 2.6)	2.0 (1.0; 3.0)	0.758
**pPCL** (mm)	1.5 (0.7; 2.2)	1.4 (0.8; 2.3)	0.862	1.4 (0.9; 2.4)	1.2 (0.7; 2.7)	0.625
**aMCL** (mm)	4.3 ± 1.4	4.1 ± 1.5	0.570	4.0 ± 1.4	3.8 ± 1.7	0.681
**iMCL** (mm)	3.8 ± 1.2	3.7 ± 1.3	0.693	3.5 ± 1.3	3.3 ± 1.4	0.693
**pMCL** (mm)	4.5 ± 1.3	4.4 ± 1.4	0.907	4.1 ± 1.5	4.0 ± 1.5	0.723
**aDMCL** (mm)	6.9 ± 2.1	6.8 ± 2.3	0.906	5.9 (4.7; 7.5)	5.4 (4.8; 7.3)	0.767
**pDMCL** (mm)	7.6 ± 2.2	7.9 ± 2.4	0.679	6.2 (5.3; 8.4)	6.5 (5.4; 7.7)	0.986
**LCL** (mm)	1.3 (1.0; 1.9)	1.7 (1.1; 2.7)	0.235	1.1 (0.8; 1.7)	1.1 (0.9; 2.0)	0.633

Values are expressed as average ± standard deviation or median (IQR) for parametric and non-parametric data respectively

Unable to genotype 1 participant for rs1138545

The rare TT (n = 1) genotype and the CT genotype were combined for the analysis

Table S17: The combined *COL1A1* rs1107946 (G/T) and *rs18000012* (G/ T) genotype effects on the absolute change in the calculated length of the anterior bundle of the ACL (aACL), posterior bundle of the ACL (pACL), anterior bundle of the PCL (aPCL), posterior bundle of the PCL (pPCL), anterior bundle of the superficial layer of the MCL (aMCL), inferior bundle of the superficial layer of the MCL (iMCL), posterior bundle of the superficial layer of the MCL (pMCL), anterior bundle of the deep layer of the MCL (aDMCL), posterior bundle of the deep layer of the MCL (pDMCL) and the LCL during internal to external tibial rotation of the uninjured dominant and non-dominant legs.

	***COL1A1* rs1107946 and rs1800012 GG Genotype** [a]	***COL1A1* rs1107946 and/or rs1800012 GT or TT Genotypes** [b]	**P-value**
	n = 27	n = 37	
**aACL** (mm)	1.1 (0.9; 2.0)	1.1 (0.9; 1.5)	0.508
**pACL** (mm)	3.3 ± 1.5	2.8 ± 1.4	0.172
**aPCL** (mm)	2.1 (1.5; 2.9)	1.9 (1.2; 3.3)	0.777
**pPCL** (mm)	1.2 (0.6; 2.2)	1.5 (0.9; 2.3)	0.171
**aMCL** (mm)	4.2 ± 1.4	4.2 ± 1.4	0.821
**iMCL** (mm)	3.7 ± 1.2	3.8 ± 1.2	0.698
**pMCL** (mm)	4.3 ± 1.2	4.5 ± 1.3	0.606
**aDMCL** (mm)	6.7 ± 2.1	6.8 ± 2.1	0.907
**pDMCL** (mm)	7.6 ± 2.2	7.7 ± 2.3	0.833
**LCL** (mm)	1.6 (1.0; 2.1)	1.4 (1.0; 1.8)	0.526

Values are expressed as average ± standard deviation or median (IQR) for parametric and non-parametric data respectively

[a] both COL1A1 variants had a GG genotype

[b] one or neither COL1A1 variants had a GG genotype

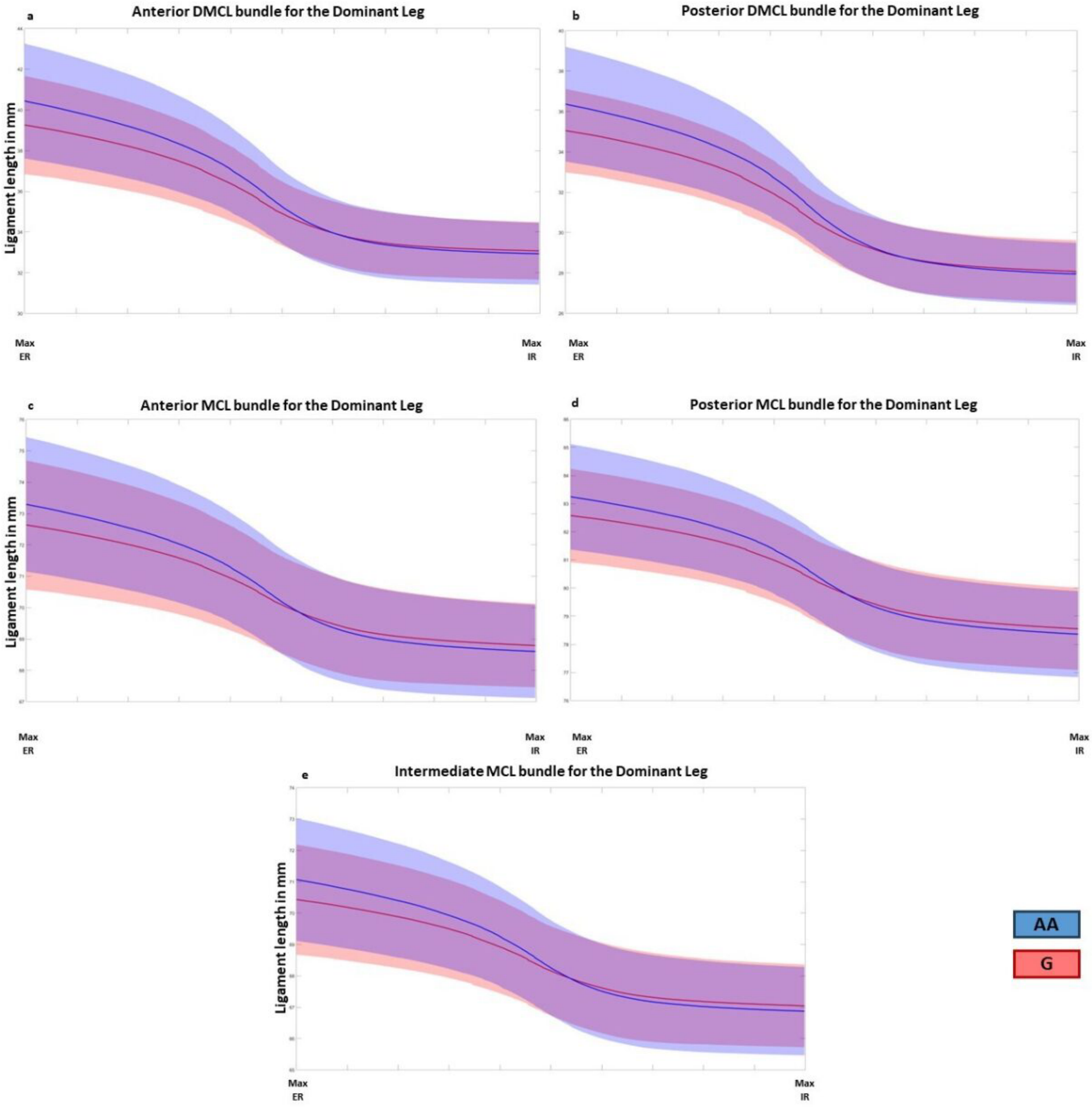

Figure S1

The *COL12A1* rs970547 (A/G) genotype effects on the five medial collateral ligament (MCL) bundle (anterior deep layer, posterior deep layer, anterior, posterior and intermediate) lengths within the dominant leg from maximum (Max) external (ER) at to maximum internal (IR) rotation. Ligament bundle lengths were calculated at every 0.02 Nm increments in applied torque during each participants' knee rotational angles from maximum external (-5 Nm torque) to maximum internal (5 Nm torque). The average (solid line) ligament length and standard deviations (shaded area) for the *COL12A1* rs970547 AA genotype (AA) and combined AG and GG genotypes (G) are should in blue and red, respectively, with areas of overlap represented in purple.

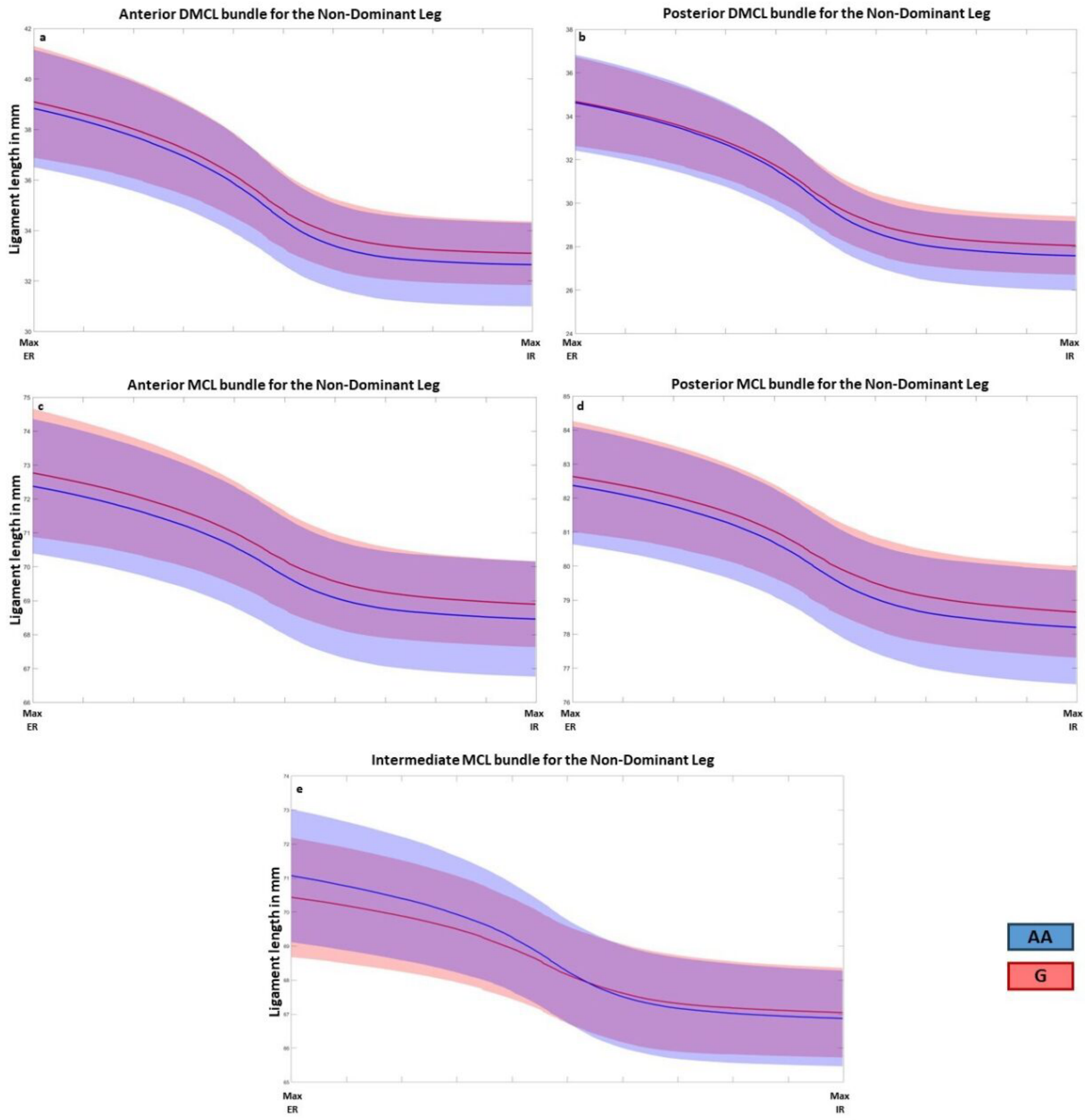

Figure S2

The *COL12A1* rs970547 (A/G) genotype effects on the five medial collateral ligament (MCL) bundle (anterior deep layer, posterior deep layer, anterior, posterior and intermediate) lengths within the non-dominant leg from maximum (Max) external (ER) at to maximum internal (IR) rotation. Ligament bundle lengths were calculated at every 0.02 Nm increments in applied torque during each participants' knee rotational angles from maximum external (-5 Nm torque) to maximum internal (5 Nm torque). The average (solid line) ligament length and standard deviations (shaded area) for the *COL12A1* rs970547 AA genotype (AA) and combined AG and GG genotypes (G) are should in blue and red, respectively, with areas of overlap represented in purple.

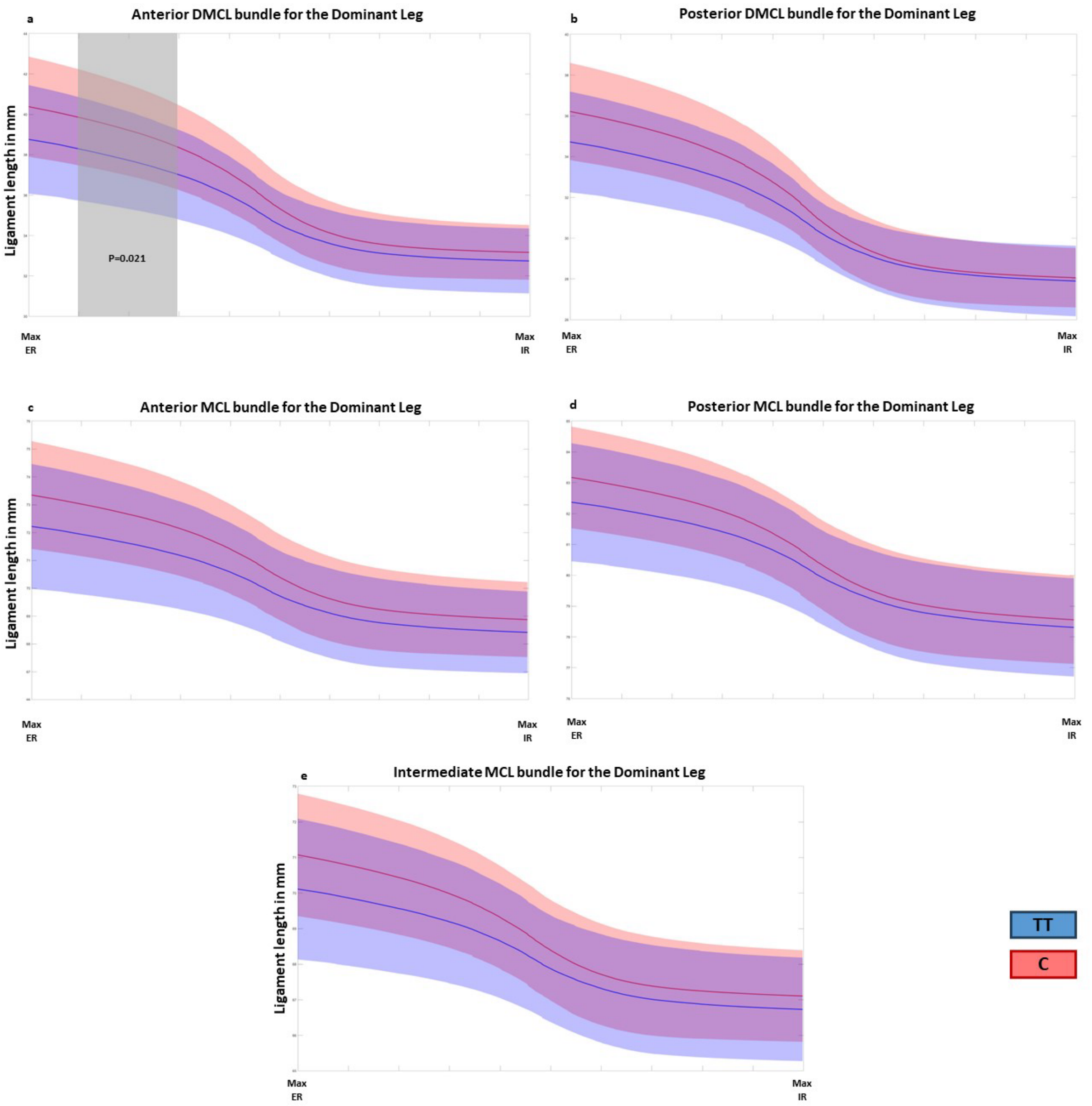

Figure S3

The *TNC* rs1061494 (T/C) genotype effects on the five medial collateral ligament (MCL) bundle (anterior deep layer, posterior deep layer, anterior, posterior and intermediate) lengths within the dominant leg from maximum (Max) external (ER) at to maximum internal (IR) rotation. Ligament bundle lengths were calculated at every 0.02 Nm increments in applied torque during each participants' knee rotational angles from maximum external (-5 Nm torque) to maximum internal (5 Nm torque). The average (solid line) ligament length and standard deviations (shaded area) for the *TNC* rs1061494 TT genotype (TT) and combined TC and CC genotypes (C) are should in blue and red, respectively, with areas of overlap represented in purple. The region of significant difference is highlighted in grey.

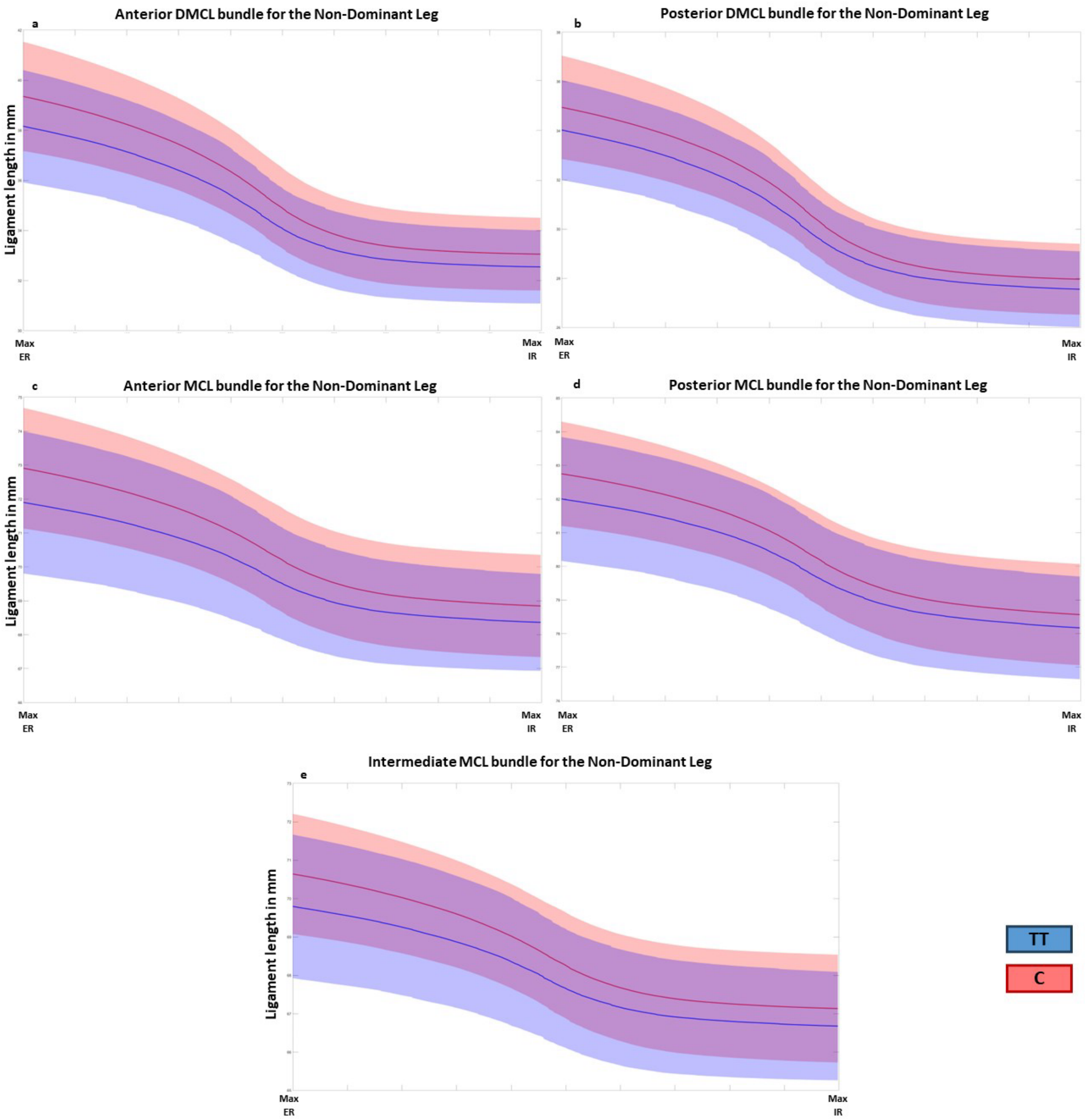

Figure S4

The *TNC* rs1061494 (T/C) genotype effects on the five medial collateral ligament (MCL) bundle (anterior deep layer, posterior deep layer, anterior, posterior and intermediate) lengths within the non-dominant leg from maximum (Max) external (ER) at to maximum internal (IR) rotation. Ligament bundle lengths were calculated at every 0.02 Nm increments in applied torque during each participants' knee rotational angles from maximum external (-5 Nm torque) to maximum internal (5 Nm torque). The average (solid line) ligament length and standard deviations (shaded area) for the *TNC* rs1061494 TT genotype (TT) and combined TC and CC genotypes (C) are should in blue and red, respectively, with areas of overlap represented in purple.

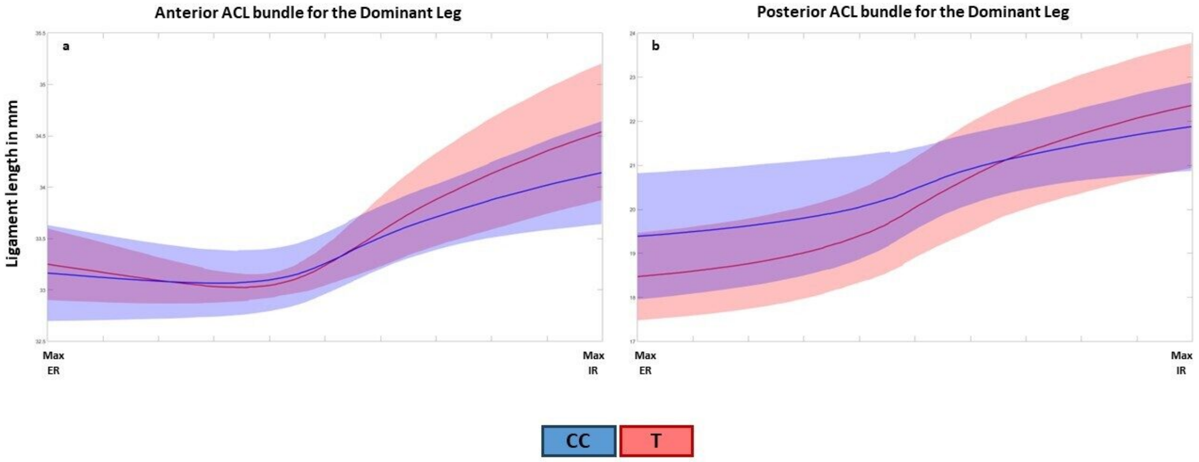

Figure S5

The *TNC* rs1061494 (T/C) genotype effects on the anterior and posterior ACL bundles lengths within the dominant leg from maximum (Max) external (ER) to maximum internal (IR) rotation. ACL bundle lengths were calculated at every 0.02 Nm increments in applied torque during each participants' knee rotational angles from maximum external (-5 Nm torque) to maximum internal (5 Nm torque). The average (solid line) ligament length and standard deviations (shaded area) for the *TNC* rs1061494 CC genotype (CC) and combined TC and TT genotypes (T) are should in blue and red, respectively, with areas of overlap represented in purple.
